# autohrf-an R package for generating data-informed event models for general linear modeling of task-based fMRI data

**DOI:** 10.3389/fnimg.2022.983324

**Published:** 2022-12-05

**Authors:** Nina Purg, Jure Demšar, Alan Anticevic, Grega Repovš

**Affiliations:** ^1^Department of Psychology, Faculty of Arts, University of Ljubljana, Ljubljana, Slovenia; ^2^Faculty of Computer and Information Science, University of Ljubljana, Ljubljana, Slovenia; ^3^Department of Psychiatry, Yale University School of Medicine, New Haven, CT, United States; ^4^Department of Psychology, Yale University School of Medicine, New Haven, CT, United States

**Keywords:** fMRI, GLM, assumed modeling, task-related activity, autohrf, R

## Abstract

The analysis of task-related fMRI data at the level of individual participants is commonly based on general linear modeling (GLM), which allows us to estimate the extent to which the BOLD signal can be explained by the task response predictors specified in the event model. The predictors are constructed by convolving the hypothesized time course of neural activity with an assumed hemodynamic response function (HRF). However, our assumptions about the components of brain activity, including their onset and duration, may be incorrect. Their timing may also differ across brain regions or from person to person, leading to inappropriate or suboptimal models, poor fit of the model to actual data, and invalid estimates of brain activity. Here, we present an approach that uses theoretically driven models of task response to define constraints on which the final model is computationally derived using actual fMRI data. Specifically, we developed autohrf–an R package that enables the evaluation and data-driven estimation of event models for GLM analysis. The highlight of the package is the automated parameter search that uses genetic algorithms to find the onset and duration of task predictors that result in the highest fitness of GLM based on the fMRI signal under predefined constraints. We evaluated the usefulness of the autohrf package on two original datasets of task-related fMRI activity, a slow event-related spatial working memory study and a mixed state-item study using the flanker task, and on a simulated slow event-related working memory data. Our results suggest that autohrf can be used to efficiently construct and evaluate better task-related brain activity models to gain a deeper understanding of BOLD task response and improve the validity of model estimates. Our study also highlights the sensitivity of fMRI analysis with GLM to precise event model specification and the need for model evaluation, especially in complex and overlapping event designs.

## 1. Introduction

fMRI remains one of the most advanced and widespread methods for studying brain function while participants perform various cognitive tasks. The standard for analyzing task-based fMRI data at the level of individual participants is using general linear modeling (GLM), which aims to explain the underlying brain activity. Predictors in the model are commonly constructed by convolving the hypothesized time course of brain activity with an assumed hemodynamic response function (HRF). In most cases, model construction is driven by predefined assumptions about the components of brain activity and their timing, which are usually based on the task design and the specific aims of the study. However, our assumptions about the onset and duration of component processes may be incorrect. They may also differ from brain region to brain region or from person to person. This may lead to inappropriate or suboptimal prediction models, poor fit of models to data, and invalid estimates of brain activity. In this paper, we present a novel approach that uses theoretical assumptions of task response to define constraints based on which the final model is computationally derived from the actual data. Specifically, we developed autohrf–an R package that enables data-driven estimation of event models for GLM analysis of task-related fMRI data. While in this paper we focus on the challenge of specifying an optimal event model across participants and regions, the approach can be extended to optimize event models for individual participants and brain regions.

The goal of task-based fMRI data analysis is to identify neural responses related to task stimuli and manipulation. In fMRI studies, neural activity is measured indirectly as the blood-oxygenation-level-dependent (BOLD) signal, which reflects changes in blood flow in response to neural activity. Neural activity and the BOLD signal are assumed to have a linear relationship that is invariant over time (Boynton et al., 1996; Cohen, 1997; Logothetis et al., 2001; Lindquist, 2008; Poldrack et al., 2011). The linearity of the relationship is reflected in the observation that changes in the BOLD signal scale linearly with changes in neural activity and neural responses to multiple task events are assumed to sum, suggesting that the BOLD signal reflects this summation of individual responses (Lindquist, 2008; Poldrack et al., 2011). This is particularly relevant for event-related study designs, where task events are presented at shorter time intervals leading to an overlap in their individual hemodynamic responses (Huettel, 2012; Liu, 2012). This linear relationship allows the use of linear statistical models that describe the time course of the BOLD signal given the expected time course of neural activity during a cognitive task, which provides the estimation of the contribution of individual responses to the measured BOLD time series.

In the GLM analysis, the BOLD signal is modeled as a convolution of task events and an assumed HRF. This approach relies on several assumptions. For instance, the model relies on prior knowledge of the experimental design and assumed cognitive processes during the task performance. In the case of most controlled studies, it is reasonable to postulate that the observed BOLD response is determined by individual responses to the stimuli as presented in the task design (Lindquist, 2008). However, it is often difficult to predict the exact number, timing, and duration of individual responses. Additionally, the model often uses a predefined HRF shape that is assumed to be stable across brain areas and individuals (e.g., Friston et al., 1994b, 1998; Boynton et al., 1996). However, studies have suggested considerable variability in hemodynamic responses between brain areas and participants (Aguirre et al., 1998; Miezin et al., 2000; Handwerker et al., 2004; Badillo et al., 2013), or related to different brain disorders (Yan et al., 2018; Rangaprakash et al., 2021). The correct choice of HRF is critical to ensure a good fit of the GLM predictors to the BOLD time series. For example, Handwerker et al. (2004) compared the performance of an assumed canonical HRF and empirically derived HRF in the detection of neural responses based on simulated BOLD time series. They have shown that empirically derived HRF gave more accurate estimates of response magnitude and higher statistical power. Therefore, the assumptions we make about neural responses significantly affect the results when using the GLM approach (Handwerker et al., 2004; Lindquist, 2008; Pernet, 2014). Even small errors in assumptions can lead to significant losses in statistical power or inflated false-positive detection (Handwerker et al., 2004; Lindquist, 2008; Loh et al., 2008). Hence, to obtain valid and accurate estimates of task response it is important to construct a model of neural activity that best fits the actual BOLD responses to neural activity.

Quite some effort has been exerted toward addressing the issue of variable HRF in task-based fMRI analysis. For example, several authors have proposed various methods for more flexible modeling of HRF that can account for different HRF shapes across brain areas and individuals (e.g., Lange and Zeger, 1997; Buxton et al., 1998; Friston et al., 1998; Glover, 1999; Lindquist and Wager, 2007; Badillo et al., 2013; Aggarwal et al., 2015; Pedregosa et al., 2015; Arias et al., 2017). Several studies have investigated the variability of HRF parameters and used that knowledge to improve the estimation of task activation (Badillo et al., 2013; Aggarwal et al., 2015; Pedregosa et al., 2015; Arias et al., 2017) or resting state functional connectivity (Rangaprakash et al., 2018; Wu et al., 2021). Nevertheless, not many studies have focused on alleviating the issue of how to specify the correct task event predictors. Some authors (Luo and Nichols, 2003; Loh et al., 2008) have attempted to develop techniques that provide a graphical representation of model fit to actual data, although these techniques still leave researchers guessing as to what would be a valid model. Because fMRI analysis involves large amounts of data, it is impractical or sometimes impossible to test the validity and fitness of the model using standard diagnostic measures (Lindquist, 2008). For these reasons, most task-based fMRI studies have not verified the validity of their event model, making their results unreliable (Lindquist, 2008). A better and more efficient approach to designing task event predictors might be to use data-informed models that are derived from the actual BOLD response. Such an approach could also take into account the variability of BOLD responses and construct separate models that fit each individual or specific brain regions.

The aim of our study was to provide a method that would allow the construction and evaluation of event models based on the actual fMRI data. Specifically, we developed the autohrf package that allows the estimation of the onset and duration of task predictors, which result in the highest fitness of GLM based on the fMRI signal under predefined constraints. We evaluated the usefulness of the autohrf package on two original datasets of task-related fMRI activity. In the first dataset (37 participants, 20 women, 19–30 years), we analyzed a slow event-related spatial working memory task. In the second dataset (30 participants, 15 women, 24–52 years), we analyzed a mixed state-item designed flanker task. The focus of our analysis was the comparison of theoretical and automatically derived models provided by the autohrf package, and the results based on them. Here, we were interested in any qualitative differences in the obtained results, in addition to changes in the fitness of the models and effect sizes of the resulting GLM estimates. We assumed that data-driven models based on the automated parameter search would provide higher fitness of the models, improve effect sizes, and give qualitatively more reliable results. We also tested generalizability of event models obtained with autohrf by performing cross-validation and investigated their validity based on simulated fMRI time series.

## 2. Methods

autohrf is available in the form of an open-source R package (licensed with GNU General Public License v3.0) and its code is stored in a public GitHub repository: https://github.com/demsarjure/autohrf. The package is also available on CRAN so users can install it *via* R's install.packages function. The core functionality of the package can be divided into two groups—functions for model evaluation and functions for automatic search for optimal event timing.

### 2.1. Evaluating pre-defined models

The evaluate_model function can be used to evaluate the fit of predefined event models to the acquired BOLD data. A predefined model is one that we define manually by specifying the onset and duration of each event within the BOLD time series under study. In R, the model is defined by storing this information in the form of a data frame:


event       start_time  duration
---------------------------------
event_1     0.00        2.50
event_2     2.50        5.00
...
event_n     10.00       3.00


To evaluate the model, we need to provide the evaluate_model function with a data frame that contains the BOLD data and the information about the BOLD sequence repetition time (TR). The BOLD data must be stored in a data frame with three columns, name of the region of interest (ROI), timestamp (t) and the value of the BOLD signal (y):


  roi     t       y
-------------------------
  ROI_1   0       2.445844
  ROI_2   0       2.155406
  ROI_3   0       2.345234
...
  ROI_n   0       2.536729
  ROI_1   2.5     7.725104
  ROI_2   2.5     4.436729
...


The evaluate_model function first generates an HRF using either the Boynton (Boynton et al., 1996) or the SPM (Statistical Parametric Mapping) method (Friston et al., 1994a, 1998). The main difference between the two methods is that the Boynton approach uses a single-gamma function to model HRF, while SPM uses a double-gamma approach. Both methods are implemented using the descriptions in the original manuscripts and parameters are set to the default values specified in the manuscripts (Boynton et al., 1996; Friston et al., 1998). However, users can fine-tune the parameters using the p_boynton and p_spm parameters.

Once the HRF is constructed, it is convolved with neural time series generated from the event descriptions. To allow high precision in the computation of the predicted BOLD time series, the neural time series and the HRF are created with a high temporal resolution, defined as *TR*/*f*. The up-sampling factor f is provided as an optional parameter with a default value of 100. After computation, the signals resulting from convolution are first down-sampled by the factor f to match the resolution of the empirical BOLD signal. Finally, to evaluate the quality of the predefined event model a linear multiple regression of the predicted BOLD time series is performed based on the empirical BOLD time series. Detailed equations used in the construction of HRF and linear regression are presented in the Supplementary material.

The function returns the fit of the model in terms of the *R*^2^ (i.e., coefficient of determination) and *BIC* (Bayesian Information Criterion). *BIC* is based on the information theory and estimates the amount of information lost by a model (McElreath, 2020). To some extent, *BIC* also takes into account the complexity of the model, so a higher *BIC* value when using a more complex model may be a sign of overfitting. It should be noted that overfitting cannot be detected by the use of information criteria alone (McElreath, 2020), but additional model comparison procedures (e.g., cross-validation) are required.

Since fitting is performed on each ROI independently, the function outputs several values—the mean, the median, the minimum, and the weighted *R*^2^ and *BIC*. The mean and median are calculated across all ROIs, while the minimum gives the value for the ROI where the model performed worst. The weighted score can be used if we want to put more weight on how the model fits a particular subset of ROIs compared to the rest of the ROIs. To accomplish this, we can specify weights for specific ROIs using the parameter roi_weights in the form of a data frame containing the name of the ROI and the weight we want to assign to it. By default, each ROI has a weight of 1. To allow for more in-depth analysis of how the model fits the data, the evaluate_model function also returns the quality of the fit for each of the used ROIs. This information is stored in the by_roi data frame that can be found within the object returned by the function. For a full list of input parameters and a description of outputs for the evaluate_model function please consult the official documentation of the autohrf package.

The quality of the fit computed with the evaluate_model function can be visually inspected using the plot_model function. The input to this function is the output of the evaluate_model function. Example visualizations resulting from the plot_model function can be found in the two study examples presented later in the paper in Sections 3 and 4.

### 2.2. Automated parameter search

The automated parameter search functionality provides the tuning of event parameters used in task-related GLM analysis in a data-driven manner. This is done using the autohrf function. Here, we start by defining the constraints of our model using the model_constraints parameter in the form of a data frame:


event       start_time  end_time
--------------------------------
event_1     0.00        2.50
event_2     2.50        6.00
...
event_n     10.00       15.00


With the above data frame, we specify that event_1 must not start before time 0 s and must end before or exactly at 2.5 s. We specify similar constraints for other events. By adding additional, optional columns to this data frame, we can impose further constraints. The first of these is called min_duration, which we can use to define the minimum duration of an event. Similarly, we can add max_duration to limit the maximum length of an event. We can specify either one such data frame or multiple data frames with different constraints. The function will then try to find the optimal model for each set of constraints independently. In this case, the function will return the best model for each of the specified sets of constraints.

Besides the model constraints, the only other two mandatory parameters of the autohrf function are the data frame containing BOLD data, which is the same as described in the previous section, and the TR. The function will then try to find an optimal model that satisfies the given constraints.

The automated parameter search in autohrf uses genetic algorithms (Holland, 1992). Genetic algorithms are a family of search algorithms that simulate natural evolution to find parameters that provide the best (or near best) solution to a given problem. The process begins with the creation of the initial population, a set of random solutions that satisfy the given constraints. The size of the population is defined by the population parameter. Once we have the starting population, the fitness of each member is evaluated using the weighted *R*^2^ score, as described in Section 2.1.

Next, a specified percentage of the best solutions, defined by the elitism parameter, is automatically copied to the next population. To maintain the same population size, the remaining solutions are generated by shuffling solutions from the current population. This involves mixing two solutions from the current population (parent) to create a new solution (child) for the next population. Solutions that have a good score (weighted by *R*^2^ in our case) have a higher probability of being selected as parents. This mechanism ensures that the parameters of good solutions have a higher probability of being propagated to the next population, which mimics the survival of the fittest in nature, where individuals with "good" genes have a higher probability of passing on their traits to subsequent generations.

**Figure 1:**
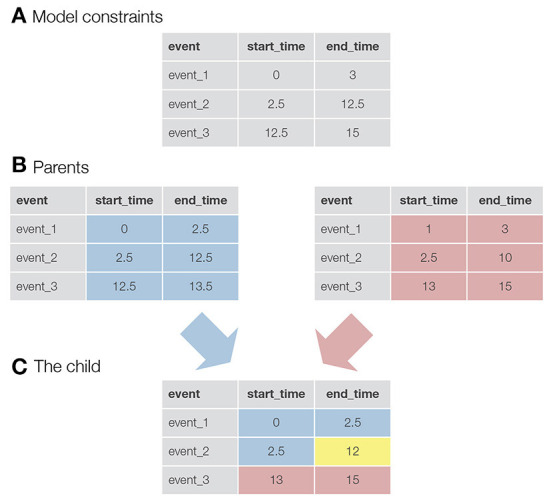
A visualization of the logic behind genetic algorithms. **(A)** An example of constraints for a model with three events. For example, the constraints for event_1 specify that the onset for this event must be greater than 0 s and the end time must be less than 3 s. **(B)** The two parents selected to produce a child for the new generation. In this case, the values in the tables are not constraints, but the actual parameters of the events. For example, event_1 starts at 0 s and ends at 2.5 s. **(C)** A newly created child. Half of the events (+1, since we have an odd number of events) and their parameters come from the first parent (colored blue), while the rest of the parameters come from the second parent (colored red). The yellow colored parameter was taken from the first parent, but here a mutation event was triggered. This event reduced the final time of event_2 from 12.5 to 12 s.

Once the parents are selected, a new child solution is generated by taking the parameters for the first half of the events from the first parent and the parameters for the second half of the events from the second parent (in the case of an odd number of events, an additional event is taken from the first parent). In addition, an event called mutation can be triggered (the exact equation used in autohrf can be found in the Supplementary material). This, in turn, mimics the natural phenomenon where genes in the DNA of species can randomly mutate and change as a result. In our case, mutation slightly changes the value of the onset or offset of an event. For each event and start/end time, there is a probability equal to the mutation_rate that a mutation event will be triggered. With mutations, we ensure that genetic algorithms not only search for the best solution within the randomly generated initial population, but also explore new solutions that may be better than those in the existing population. See Figure 1 for a visualization of how two parent solutions are used to generate a new child solution.

The process of parent selection and child creation repeats until the new population size equals the value of the population parameter. Once they are equal, we have a new population and the first iteration of the process is complete. Next, the entire process is repeated—each solution is evaluated, the best solutions are copied to the new population, the rest are generated by the child creation process, and so on. We repeat the process until we reach the number of iterations specified by the iter parameter. For a full list of input parameters and a description of outputs for the autohrf function please consult the official documentation of the autohrf package.

**Figure 2:**
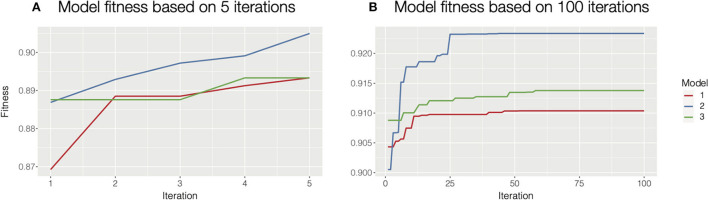
Diagnosing model fitness with plot_fitness function. In this case, we used three different model constraints, hence we computed three models. **(A)** For illustrative purposes we ran autohrf for only 5 iterations with a population of 10. It is clear that the solutions at the end did not converge as the fitness is still rising. In such cases, we should increase the number of iterations or the population size and repeat the process. We could also rerun autohrf and set its autohrf parameter to the outputs of the previous run, which will continue the automated parameter search by using the results of the previous run. **(B)** In this case, we ran autohrf with the same model constraints, but this time we used the default settings (100 iterations with a population size of 100). It is clear that the solutions here converged as the optimal solution did not change for any of our constraints in the last 40 iterations.

Besides the autohrf function, there are additional helper functions that make the process more user-friendly. The plot_fitness function should be used as a diagnostic tool to check if the solutions have converged. The function plots how the fitness (the weighted *R*^2^) of the best model changes through iterations for each of the specified model constraints. Given a sufficient number of iterations, the fitting will always converge, ideally to the global maximum. Increasing the population size or mutation rate will help in cases where the process is trapped in a local maximum. The only input parameter for this function is the output of the autohrf function. See Figure 2 for an illustration of how plot_fitness can be used to investigate whether the autohrf run converged.

The plot_best_models function is used to plot the best model for each of the given constraints. Example visualizations for this function can be found in the study examples in Sections 3 and 4.

The get_best_models function returns and prints the parameters of the best model for each of the specified model constraints. The output of this function can be used for further analysis and also as an input to the function for evaluating predefined models. By setting the return_fitness parameter to TRUE, the function will not return parameters of the best models but their fitness.

## 3. Spatial working memory task

To test and evaluate the utility of the autohrf package, we used the data collected in a spatial working memory study, previously presented in Purg et al. (2022). The aim of the study was to investigate neural correlates of different spatial working memory strategies and related representations that are used depending on specific task demands. We used fMRI to record brain activity during the task performance.

To estimate the activity on individual task trials, we used a slow event-related study design, which allowed enough time for the BOLD response to return to baseline during an inter-trial interval (ITI). However, each trial consisted of several events that were assumed to trigger overlapping hemodynamic responses. We employed the GLM approach to differentiate between these individual responses. The challenge that we faced in constructing the event model to be used in GLM analysis was specifying the number, timing and duration of individual events that would result in valid estimates of brain responses to task events of interest. For that purpose, we used autohrf to evaluate different theoretical event models and to provide additional insights into the properties of BOLD responses during the task using event models optimized in an automated data-driven process.

In this paper, we present an example procedure of selecting the most appropriate model using autohrf, while simultaneously evaluating the generalizability of event models obtained with autohrf and the reliability of their results. Additionally, we test the validity of obtained event parameters based on simulated fMRI time series.

### 3.1. Data information

The analysis included 37 healthy adults (20 women, *M* = 21 years, *SD* = 3 years, range = 19–30 years). The study was approved by the Ethics Committee of the Faculty of Arts, University of Ljubljana, Slovenia, and the National Medical Ethics Committee, Ministry of Health of the Republic of Slovenia. Participants gave written informed consent before participating in the study.

**Figure 3:**
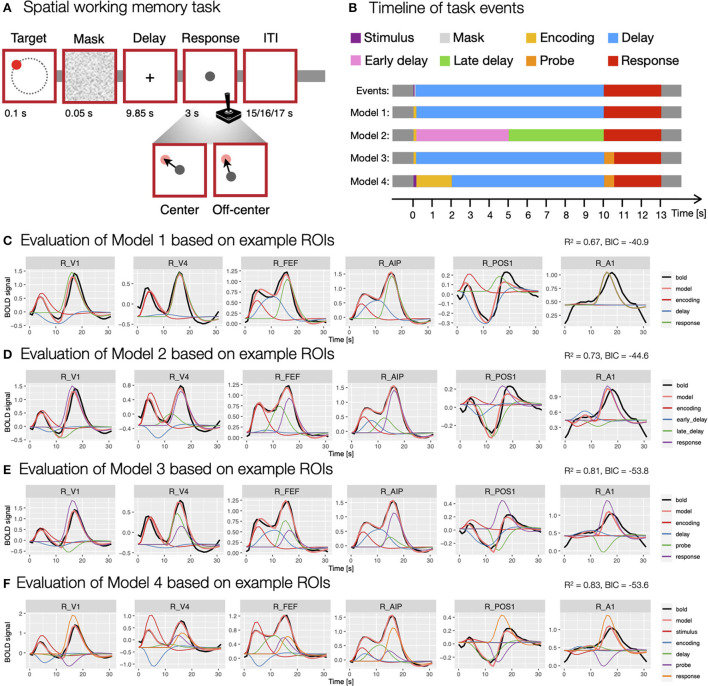
Modeling of the spatial working memory task trial using different event models. **(A)** The design of the spatial working memory task. Participants were asked to remember the position of a briefly presented target stimulus and, after a delay period, to give a response by moving a probe using a joystick to the previously remembered target position either from the center of the screen (*center* condition) or a random off-center position (*off-center* condition). **(B)** The timing and duration of different assumed events in the task, which were aligned to TR = 1 s used in the fMRI recording. **(C–F)** The evaluation of specific models. The models were evaluated using the evaluate_model function with double-gamma HRF based on the average activity during a task trial for individual ROIs. The individual plots were prepared using the plot_model based on the activity in six example ROIs (i.e., R_V1, right primary visual cortex; R_V4, right fourth visual area; R_FEF, right frontal eye fields; R_AIP, right anterior intraparietal area; R_POS1, right parieto-occipital sulcus area 1; R_A1, right primary auditory cortex; Glasser et al., 2016) that differed in BOLD responses during a spatial working memory task. The black line shows the average BOLD response, the pink line the modeled BOLD response and the thin colored lines depict individual responses to specific events. The reported *R*^2^ and *BIC* represent mean values across all 360 ROIs in the HCP-MMP1.0 parcellation (Glasser et al., 2016).

In the spatial working memory task (Figure 3A), each trial began with a presentation of a target stimulus (red disk) for 0.1 s on a screen with a constant radius and at different angles from the center of the screen, followed by a 0.05 s masking pattern. Participants were asked to remember the position of the target stimulus and to maintain it during the following 9.85 s delay period. After the delay period, participants responded by moving a probe (gray disk) to the position of the remembered target using a joystick. The response time was fixed, such that the position of the probe after 3 s was recorded as the response position. Individual trials were separated by ITIs whose duration varied randomly (15, 16 or 17 s with a ratio of 3:2:1) to allow for better decomposition of task-related BOLD responses. The task consisted of two conditions—*center* and *off-center*. In the *center* condition, the response probe always appeared at the center of the screen, whereas it appeared off-center with a constant radius but at a random angle to the target position in the *off-center* condition. The two task conditions were designed to differentiate between different spatial working memory strategies (for a detailed description of the task see Purg et al., 2022).

Brain activity during the task performance was measured with simultaneous fMRI and EEG recording in one to three recording sessions (6 participants with one session, 12 participants with two sessions, and 19 participants with three sessions). Consecutive sessions of the same participant were separated on average by five weeks (range = 2–13 weeks). In this paper, we report results based only on fMRI data. Data from participants who attended multiple sessions were combined across sessions. Each task condition was performed in a separate BOLD run with 24 trials within individual sessions. The order of the task conditions was pseudorandomly varied in the first session and counterbalanced across participants. The order of task conditions was then reversed for each subsequent session of the same participant. MRI data were collected with Philips Achieva 3.0T TX scanner. T1- and T2-weighted structural images, spin-echo field map images and BOLD images were acquired for each participant. Detailed MRI acquisition parameters are described in the Supplementary material.

### 3.2. Data analysis

MRI preprocessing and analysis were performed using Quantitative Neuroimaging Environment & Toolbox (QuNex, v0.94.14; Ji et al., 2022). Parts of the analysis and visualizations were prepared using R (v4.2.1; R Core Team, 2022) and Connectome Workbench (v1.5.0) tools. MRI data were preprocessed with the Human Connectome Project (HCP) minimal preprocessing pipelines (Glasser et al., 2013 for details see the Supplementary material). Further analyses were performed on parcellated whole-brain data, which were obtained by extracting the mean signal for each ROI as identified in the HCP-MMP1.0 parcellation (Glasser et al., 2016).

We performed the activation analysis using a GLM approach in which event predictors were convolved with the assumed double-gamma HRF (Friston et al., 1994a, 1998). We prepared several models that were either based on theoretical assumptions of the number and timing of processes in the task or automatically derived based on the actual BOLD response under predefined restrictions using the autohrf package. Using these models, we then modeled the BOLD signal during each task condition, separately for each participant, and estimated *β* coefficients for each task event predictor in the model. We also modeled trials with outlier behavioral responses as a separate event in the GLM and excluded these trials from further analysis. Statistical analyses at the group level were performed based on the obtained *β* estimates using permutation analysis (500 permutations, tail acceleration) in PALM (Winkler et al., 2014). To identify activation and deactivation during individual events, we performed two-tailed one-sample *t*-tests with FDR multiple comparison correction. We also directly compared the two task conditions by performing two-tailed paired *t*-tests with FDR correction. The significance threshold was set at *q* < 0.05.

For the cross-validation of models derived using autohrf, we used the same GLM approach as described earlier, but this time each fMRI recording session was modeled separately. Based on the obtained *β* estimates we then performed a hold-out cross-validation based on 31 participants that attended at least two recording sessions, among them there were 19 participants with three recording sessions. The fMRI data obtained at the first session were used for the training of the models using the autohrf function, while the data from the second and third sessions were used to test the models using the evaluate_model function.

We additionally tested autohrf on simulated fMRI data. We generated simulated BOLD responses to a working memory trial based on four different event models. All four models included the same number and type of event predictors, but differed in the specific onset and duration of the events. They were based either on theoretical assumptions about processes in the spatial working memory task (Models A and B; Supplementary Figure S1) or on autohrf estimates based on empirical data (Models C and D; Supplementary Figure S1). For each model, a set of BOLD time series was generated by convolving the event time series with an assumed double-gamma HRF (Figure 6A). In generating the data, we systematically varied the HRF properties and noise level. We used HRF with 4 s, 5 s, and 6 s time-to-peak. This included the autohrf default value of 5 s and roughly covered the range of empirically measured HRFs (Aguirre et al., 1998; Miezin et al., 2000; Handwerker et al., 2004). We generated data at five different noise levels by adding time series of random Gaussian noise with different SD values (i.e., 0, 0.067, 0.1, 0.15, 0.225) to the generated BOLD signal. For each of the described combinations of event model, HRF, and noise level, data time series were generated for 10 hypothetical ROIs that differed in the amplitudes of modeled responses to specific events. Next, we performed automatic parameter search for each of the 60 simulated datasets with two sets of predefined constraints, a strict model and a more permissive model. We evaluated the results obtained with autohrf in two ways. First, we compared the estimated event models with the simulated models to evaluate the extent to which autohrf identified the underlying event structure. Second, we generated new synthetic data with a larger set of 216 simulated ROIs and then compared the *β* estimates obtained with the two theoretical and two autohrf-optimized models with the values used to generate the data (see Supplementary material for details on the data simulation).

### 3.3. Results

#### 3.3.1. The comparison of models with different number of event predictors

In the analysis of BOLD signal underlying the performance of the spatial working memory task, we were faced with the challenge of choosing an event model that would fit our data well and provide valid estimates of brain responses to individual task events. In the construction of the most appropriate model, we were first faced with the question of how many and which task events we should include in the model. To that end, we prepared and evaluated several models containing different types and numbers of events (Figure 3B). To evaluate models, we used the evaluate_model function with the double-gamma HRF based on the average activity during a task trial for individual ROIs.

We started the analysis with the most simple model consisting of three events (Model 1; Figure 3C), the *encoding* (onset = 0 s, duration = 0.15 s), *delay* (onset = 0.15 s, duration = 9.85 s) and *response* (onset = 10 s, duration = 3 s). To investigate changes during the delay period of the task, we prepared a separate model (Model 2; Figure 3D) where the delay period was separated into two events, an *early delay* (onset = 0.15 s, duration = 4.85 s) and a *late delay* (onset = 5 s, duration = 5 s). Additionally, we took into account that the encoding and response phases include a progression of several individual processes. For example, the response period of the task starts with the presentation of a probe stimulus which is assumed to trigger sensory and attentional processes, followed by motor-related activity of the required hand response. To reflect that, we constructed a model that included *encoding* (onset = 0 s, duration = 0.15 s), *delay* (onset = 0.15 s, duration = 9.85 s), *probe* (onset = 10 s, duration = 0.5 s) and *response* (onset = 10.5 s, duration = 2.5 s) events (Model 3; Figure 3E). Similarly, the encoding phase can be split into temporally separated events for the initial sensory processing of the target stimulus and a following encoding process. To address this possibility, we included a model with *stimulus* (onset = 0 s, duration = 0.15 s), *encoding* (onset = 0.15 s, duration = 1.85 s), *delay* (onset = 2 s, duration = 8 s), *probe* (onset = 10 s, duration = 0.5 s) and *response* (onset = 10.5 s, duration = 2.5 s) events (Model 4; Figure 3F).

The comparison of these models revealed considerable differences in the fitness of the models, as well as the obtained estimates of individual responses. An increase in the number of events resulted in a higher mean fitness, with the worst fitness observed in the model with three events (*R*^2^ = 0.67, Figure 3C) and the highest in the model with five events (*R*^2^ = 0.83, Figure 3F). This result was expected since a model with more predictors offers more degrees of freedom in modeling and is able to explain more data variance. However, models with a higher number of predictors can lead to data overfitting and poor generalizability. To avoid that, we also compared *BIC* values between models, where relatively higher values in more complex models may indicate a possibility of overfitting. Our results showed decreasing *BIC* values from Model 1 to 3 (Figures 3C–E), whereas the *BIC* measure increased at the Model 4 (Figure 3F), suggesting that separating encoding and response periods into two phases may lead to overfitting.

Additionally, qualitative inspection of the obtained estimates showed generally less valid results with increasing number of events. For example, the visualization of GLM estimates based on example ROIs (Figures 3C–F) sometimes revealed counterintuitive estimates, such as negative *β* estimates during positive BOLD responses. In addition, individual responses sometimes appeared to be overestimated or underestimated with increasing number of events. The results suggested that additional regressors primarily addressed the variability in the HRF shape, rather than providing insight into temporally separable events. We concluded that the apparent reduction of the validity of *β* estimates offsets the increased fitness of more complex models. For that reason, we decided to use the first two models (i.e., Model 1 and 2; Figure 3B) for further analysis, as they exhibited sufficient fitness and produced *β* estimates with highest face validity for the task events of interest.

#### 3.3.2. The comparison of theoretically and automatically derived event models

Next, we wanted to determine the optimal timing of individual task event predictors for the chosen models. For example, we were unsure when the encoding phase ends and the delay phase starts, how soon after the delay period the response phase starts, how long are individual processes in the task, etc. For that purpose, we used the autohrf package to compute and evaluate different onsets and durations of events in the models based on theoretical assumptions of the event time course in the task or using the automated parameter search based on the actual fMRI data. Particularly, we compared the fitness of theoretical and automatically derived models, along with the obtained effect sizes and any qualitative changes in the results obtained with each model.

**Figure 4:**
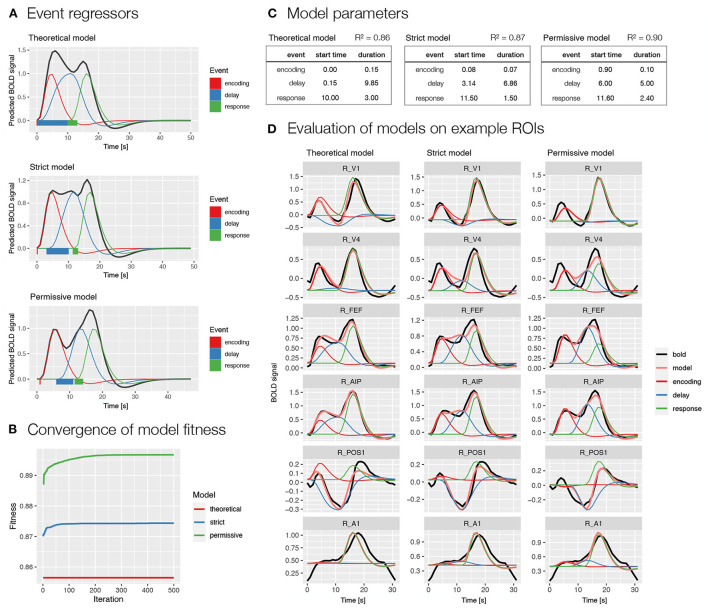
Specification and performance of theoretical and automatically derived event models in the spatial working memory study. Here, we compare three models differentiating between *encoding, delay* and *response* events, a theoretically derived model based on the assumed timeline of events in the task and two automatically derived models based on the empirical BOLD signal using the autohrf function that differed in the predefined constraints, the permissive model was allowed larger deviations from the theoretical model than the strict model. The models were fit to the activity of a predefined selection of 80 ROIs in the frontoparietal network, in addition to visual and motor-related brain areas that were assumed to show responses to the task. **(A)** A visualization of task event predictors convolved with double-gamma HRF obtained using the plot_best_models, where the colored lines depict individual responses to specific events and the black line shows the summation of these responses in the BOLD signal. Rectangles at the bottom visualize the onset and duration for each of the events in the model. **(B)** The convergence of the model fitness in the automated parameter search based on the population of 100 and 500 iterations. The plot was obtained using the plot_fitness. **(C)** Theoretical and data-driven onset and duration of task events in the models. The *R*^2^ shows the mean fitness of the models across the selected 80 ROIs. **(D)** The evaluation of the models on six example ROIs (i.e., R_V1, right primary visual cortex; R_V4, right fourth visual area; R_FEF, right frontal eye fields; R_AIP, right anterior intraparietal area; R_POS1, right parieto-occipital sulcus area 1; R_A1, right primary auditory cortex; Glasser et al., 2016) with different types of BOLD response during a spatial working memory task. The plots were prepared using the plot_model, where the black line shows the average BOLD response, the pink line the modeled BOLD response and the thin colored lines depict individual responses to specific events.

We used two models selected based on the previous analysis (i.e., Model 1 and 2; Figure 3B). Specifically, we first modeled BOLD signal with events *encoding, delay*, and *response* (Figure 4), whereas in the second model we additionally modeled the delay phase with separate regressors for *early* and *late delay* (Supplementary Figure S3). For each of the two models, we first evaluated the event timing that was defined based on theoretical assumptions. Since the theoretical assumptions might not match with actual processes in the task, we also prepared two models with automatically derived event onset and duration based on the BOLD signal using the autohrf function, one with stricter predefined restrictions and one with looser predefined restrictions. The stricter model allowed an automatic adjustment of the event duration, while the event onset and offset boundaries were kept the same as in the theoretical model. In the more permissive model, the event onset and offset settings allowed a deviation from the theoretical model for 1 s in each direction, while the duration was set to reach at least a half of the theoretically assumed duration. We ran the automated parameter search on a predefined selection of 80 ROIs in the frontoparietal network, visual, and motor-related brain areas that were expected to show responses to the task.

The comparison of theoretically and automatically derived models showed better model fit for the automatic compared to theoretical models (Figures 4B,C and Supplementary Figures S3B,C). The automatically derived onset and duration of events differed considerably from the theoretical parameters. Specifically, the onset of all events was generally estimated at later times in the automatic compared to theoretical models, whereas the duration of events was generally decreased (Figures 4A,C and Supplementary Figures S3A,C). A qualitative inspection of the obtained *β* estimates of individual responses on example ROIs (Figure 4D and Supplementary Figure S3D) indicated estimates with high face validity for the encoding and response phases across all models, whereas the delay estimates appeared to be slightly overestimated in the automatic models, especially in the case of a single *delay* predictor (Figure 4D).

**Figure 5:**
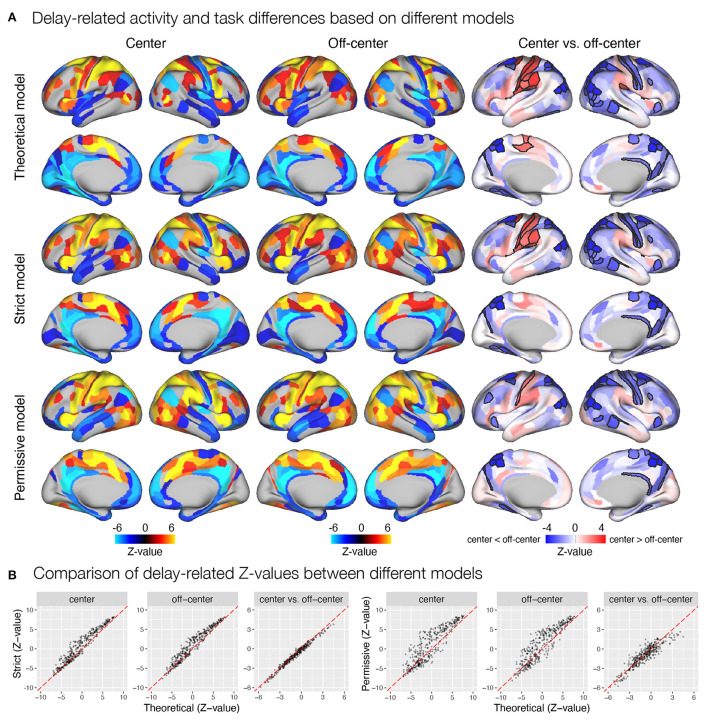
Delay-related activity and differences between task conditions estimated based on different event models in the spatial working memory study. Results were obtained by modeling *encoding, delay*, and *response*, with the timing and duration of each event either theoretically determined or derived automatically based on the autohrf using less or more permissive constraints. **(A)** Results of the statistical analysis show delay-related activity separately for the *center* and *off-center* conditions and delay-related activity differences between task conditions. The results of activation and deactivation during individual task conditions (the first two columns) show only statistically significant results at *q* < 0.05. On the other hand, the task differences (the third column) are presented across all ROIs, while the black outlines mark statistical significance at *q* < 0.05. **(B)** The comparison of *Z*-values of individual ROIs between different models. Each dot represents the *Z*-value for a single parcel obtained with the contrasted models. The dashed red line represents the diagonal.

We were also interested in any differences in the results of statistical analyses based on *β* estimates obtained from theoretical and automatically derived models. Specifically, we examined significant activation and deactivation during individual task events modeled in the GLM analysis, separately for the *center* and *off-center* task conditions. We also contrasted *β* estimates between *center* and *off-center* conditions to identify any significant differences in the observed responses. The models indeed resulted in slightly different patterns of identified task-related (de)activations and differences between task conditions. For instance, the estimates of delay-related activity obtained using the automatically derived event models were generally higher in the somatomotor and early visual areas, and lower in the temporal brain regions compared to the estimates obtained with the theoretically based event model (Figure 5A). Further, contrasting of task conditions revealed less ROIs with a higher activity for the *center* compared to the *off-center* condition in the somatomotor areas, and additional ROIs in the prefrontal and insular cortices showing higher activity for the *off-center* condition when using automatically derived compared to theoretically based event models. Similar qualitative differences were observed also during encoding (Supplementary Figures S4A, S6A) and response (Supplementary Figures S5A, S9A) phases, in addition to the two separate estimates for the delay-period activity, early (Supplementary Figure S7A) and late delay (Supplementary Figure S8A). We additionally compared *Z*-values for different brain maps between theoretical and automatically derived models, which showed substantial differences in *Z*-values that were highly variable between specific comparisons (Figure 5B and Supplementary Figures S4B–9B). In general, *Z*-values tended to be increased for the results based on automatically derived event models.

#### 3.3.3. The generalizability of automatically obtained models across different fMRI recording sessions

To test the generalizability of models obtained with autohrf we performed a hold-out cross-validation using fMRI data collected at different recording sessions for 31 participants with two or three sessions. Specifically, we tested two models, one with stricter predefined restrictions and one with looser predefined restrictions, as described in Section 3.3.2. The fMRI data obtained at the first session were used for the training of the models using the autohrf function, while the data from the second and third sessions were used to test the models using the evaluate_model function. Both models were trained and tested based on a predefined selection of 80 ROIs that were expected to show responses to the task. During the model training (Session 1; Supplementary Figure S10), the permissive model showed a higher mean fitness (*R*^2^ = 0.89) compared to the strict model (*R*^2^ = 0.87). In comparison, the mean fitness of the theoretical model on the same dataset was *R*^2^ = 0.86. During the evaluation of the models based on the data from the second and third recording sessions, the mean model fitness was again the highest for the permissive model (Session 2: *R*^2^ = 0.88; Session 3: *R*^2^ = 0.89), second highest for the strict model (Session 2: *R*^2^ = 0.85; Session 3: *R*^2^ = 0.86), and the lowest for the theoretical model (Session 2: *R*^2^ = 0.84; Session 3: *R*^2^ = 0.85). Since the model fitness remained similar even during the testing compared to the training of the models, these results suggest high generalizability of models obtained using autohrf.

Next, we were also interested in the consistency of event parameters when the autohrf function is given the same set of predefined constraints, but the model is trained based on different datasets. For that purpose, we used the data from 19 participants with three recording sessions and ran an automatic parameter search using strict and permissive restrictions based on each separate recording session. Our results showed generally very consistent event onset and duration across sessions for both, strict and permissive, models (Supplementary Figure S11). To quantitatively estimate the consistency of event parameters across sessions, we calculated the percentage of overlap in event timing during a task trial across sessions in relation to the total time interval covered by the event in at least one of the sessions, separately for *encoding, delay*, and *response* events (Supplementary Figure S11B). For the strict model, we observed a 100% overlap for the *encoding*, 89% for the *delay*, and 87% for the *response*. Similarly, the permissive model returned an overlap across sessions of 49% for the *encoding*, 100% for the *delay*, and 79% for the *response*.

#### 3.3.4. The evaluation of automatically obtained event models based on simulated fMRI data

Even though our results suggest good reliability and generalizability of event models obtained with autohrf, the problem with evaluating the validity of event models based on empirical fMRI data is that we do not know what kind of cognitive processes actually gave rise to the measured BOLD signal and thus cannot be sure that the obtained event parameters are valid. This can be addressed by running autohrf on simulated fMRI data with known event parameters that are used to generate the BOLD signal. Additionally, there are other factors that may influence the validity of estimates obtained with autohrf. For instance, we were interested to what extent different noise levels interfere with reliable estimates. Moreover, since the automated parameter search in autohrf uses predefined and fixed HRF timing, we wanted to check the sensitivity of autohrf results to the variability in the HRF timing in the measured BOLD signal.

**Figure 6:**
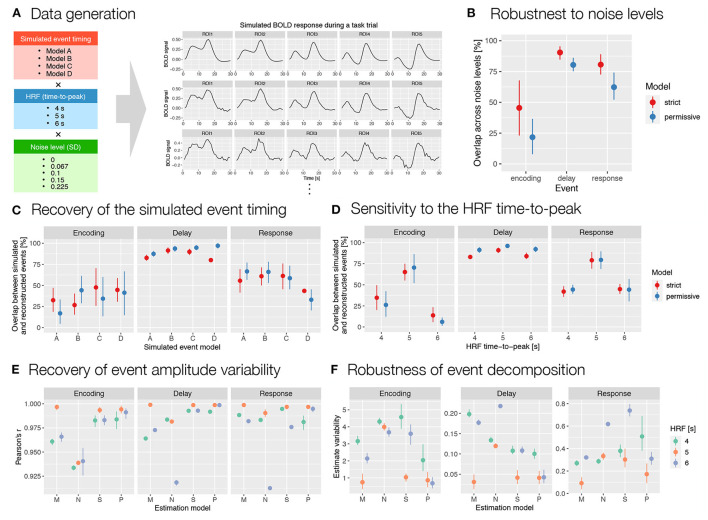
Evaluation of the results obtained with autohrf based on simulated working memory fMRI data. **(A)** Simulated BOLD signal during a working memory task trial was computed as a convolution of predicted event time series and a double-gamma HRF. We prepared 60 simulations that differed in the predicted event timing (*encoding, delay*, and *response* events with different onset and durations), HRF time-to-peak, and noise levels (i.e., added random Gaussian noise with different SD values). Each simulation was prepared based on 10 ROIs (only 5 are shown for schematic purposes) with different event amplitudes. We then ran the automated parameter search using autohrf with either strict or permissive constraints based on simulated BOLD signal. **(B)** The robustness of data-driven autohrf models to noise levels. The overlap was computed as the percentage of time covered by the automatically obtained event parameter across all noise levels, divided by the entire time range that included this event in at least one of five noise simulations, separately for each simulated event model and HRF timing. **(C)** The ability of autohrf to recover the simulated event timing based on four different models. The overlap was calculated as the percentage of the simulated event parameter covered by the automatically estimated event parameter. **(D)** The sensitivity of autohrf to differences in HRF time-to-peak. The overlap was calculated as the percentage of the simulated event parameter covered by the automatically estimated event parameter. **(E)** Pearson's correlations between simulated event amplitudes and estimated *β* values using different estimation models (M–theoretical event model matching the simulated event model, N–theoretical event model not matching the simulated event model, S–autohrf-optimized model using strict constraints, P–autohrf-optimized model using permissive constraints). **(F)** The range of *β* estimates across ROIs in which the simulated event amplitude was constant when using different estimation models. The points show the means and the ranges show the 95% confidence intervals computed with bootstrapping.

In analyzing and evaluating the simulation results, we first focused on the reconstructed timing and duration of the events. To assess the robustness to noise, we calculated the relative overlap between event reconstructions at different noise levels for each combination of simulated event models and HRF timings. The results showed relatively high overlap for the *delay* and *response* events and moderate overlap for the *encoding* (Figure 6B). To assess the accuracy of the reconstructed timing, we then calculated the percent overlap of the reconstructed events with the simulated events for all simulated event models, HRFs and noise levels (Figure 6C). The overlap was highest for the longest event (i.e., *delay*) and smallest and most variable for the shortest event (i.e., *encoding*). This is to be expected, since for shorter events even the smallest change in timing leads to large changes in overlap. Finally, to assess the sensitivity of autohrf to differences in HRF timing, we compared the overlap between the simulated and estimated events for different HRF times-to-peak. The results showed the highest overlap for the 5 s HRF time-to-peak, which was matched with the default HRF timing in autohrf, and lower overlap for the unmatched HRF times-to-peak (Figure 6D). Again, the differences in relative overlap were more pronounced for shorter events than for longer ones. A detailed review of the actual reconstructed timing (Supplementary Figure S12) shows that autohrf adjusted the timing to compensate for the differences in HRF time-to-peak. In models with longer HRF time-to-peak, events were estimated to start later. In models with shorter HRF time-to-peak, events were estimated to start earlier and to be shorter. This should allow more precise estimation of brain activity in the cases of a mismatch between the assumed and measured HRF timing.

Next, we focused on evaluating *β* estimates. We used datasets with a larger number of simulated ROIs (see the Supplementary material) to compare the ability of the two theoretical models and the models optimized with autohrf to recover simulated activity. We evaluated and compared the obtained *β* estimates in two ways. First, we calculated Pearson's *r* to test the extent to which each linear model reproduced the variability in activation of the three simulated events. The optimized models performed better than the theoretical models in most cases (Figure 6E and Supplementary Figure S13A). Their advantage was evident in the cases where the theoretical model was misspecified and where the HRFs did not match. In both cases, the optimized models fitted the data better and reproduced the underlying variability of activation better. The performance of the optimized models was again better for events of longer duration (i.e., *delay* and *response*) than for very short events (i.e., *encoding*). Second, we examined the extent to which the models provided an unbiased estimate of individual event-related activations. Specifically, we calculated the range of estimated values for all simulated ROIs in which the simulated activity of the event under study was the same but the activations of other events were different. In other words, we examined the extent to which the *β* estimate for the target event (e.g., delay) was affected by the activity related to the preceding (e.g., *encoding*) or subsequent event (e.g., *response*). Results generally showed better performance of the optimized models (Figure 6F). The performance differed depending on the specific event structures simulated and the event studied (Supplementary Figure S13B). While the advantages of the optimized models were clear for *delay*, they were less clear for *encoding* and *response* events.

## 4. The flanker task

The second illustration of the utility of the autohrf package is the analysis of a flanker task. In this study (Ličen et al., in preparation), we were interested in the extent to which different types of incentives affect performance on the flanker task and its neural correlates. To obtain separate estimates of the effect of incentive on sustained cognitive processes (e.g., goal representation, attention) and transient responses to congruent and incongruent stimuli, we used a mixed state-item design in which incentives were manipulated across different task blocks, each block consisting of a pseudorandom mixture of congruent and incongruent trials.

In the analysis of state-item designs, regressors for sustained activity and for transient responses are included in the GLM simultaneously. To obtain valid estimates of the overlapping regressors, it is important to model both the sustained and transient responses as accurately as possible because any unexplained variance from one regressor will affect the other. To avoid the problem of suboptimal modeling of the transient responses using the assumed HRF, one possible solution is to model the transient responses using unassumed modeling, where we model each time point of the BOLD response separately.

In our task, we faced an additional challenge. Reaction times in the baseline and incentive conditions differed significantly (719 and 557 ms for the incongruent and congruent trials in the baseline condition, respectively, and 600 and 464 ms for the incentive condition), so the observed differences in transient response may be due to the time-on-task effect rather than differences in neural activity. The magnitude of the time-on-task effect can be estimated and controlled by including an additional regressor in the GLM in which each event is scaled by its reaction time. The problem with this solution is that reaction times differ not only between congruent and incongruent conditions, but also between baseline and incentive blocks. Because reaction times were shorter in the incentive blocks than in the baseline blocks, differences in sustained activity between blocks could be incorrectly attributed to the transient response reaction time regressor, leading to invalid estimates of sustained activity (i.e., underestimation of differences between baseline and incentive blocks).

To address the above challenges, we adopted a two-step approach. In the first step, our goal was to obtain optimal estimates of the assumed sustained responses by fully capturing the transient responses using unassumed modeling. In the second step, we focused on decoupling activity differences and the time-on-task effect in the transient responses. Since the validity of the estimates of both sustained activity and transient responses in the proposed two-step approach is highly dependent on how well the sustained response is modeled across task blocks, we used autohrf to define the optimal event specification for modeling task block related BOLD response. The paper presenting the specific research questions addressed with this dataset and the obtained findings is currently in preparation (Ličen et al., in preparation). Rather than address specific research questions, in this section we focus only on the use of the autohrf package to optimize the event model for the study.

### 4.1. Data information

The analysis included data from 30 healthy adults (15 women, *M* = 37 years, *SD* = 8 years, range = 24–52 years) who provided written informed consent to participate in the study. The study was approved by the Ethics Committee of the Faculty of Arts, University of Ljubljana, Slovenia.

Each participant completed three BOLD runs of the Eriksen flanker task (Eriksen and Eriksen, 1974). Each run consisted of four 60 s task blocks separated by 10 s rest periods. Each task block began with the appearance of a colored rectangle surrounding the center of the screen indicating the start of the task and the incentive condition. The first trial followed 2.5 or 5 s after box onset. Each trial began with a presentation of a target stimulus (500 ms), a set of seven arrows ("<") displayed in the center of the screen. The arrows either pointed in the same direction ("<<<<<<<"; a congruent trial) or the middle arrow pointed in the opposite direction (">>><>>>"; an incongruent trial). The participant's task was to indicate by a button press as quickly as possible the direction in which the middle arrow was pointing. The stimulus display was followed by a 1.5 s response period that ended with the presentation of a feedback. The feedback was provided by a small box presented at the center of the screen for 300 ms, which was white when no response was provided and red or blue when the response was incorrect or correct, respectively. Reaction time and accuracy were recorded for each trial. To support analysis of transient responses, the ITI was jittered and lasted 0.2, 2.7, or 5.2 s in a 9:6:4 ratio. After the last ITI of the block, the colored rectangle disappeared, signaling the beginning of a rest period. Each block consisted of 13 trials presented in a counterbalanced pseudorandom order such that the same number of left and right responses and congruent and incongruent trials occurred across all four blocks.

MRI data were acquired with Philips Achieva 3.0T. For each participant we collected structural T1 and T2-weighted high-resolution images, whole-brain functional scans with a T2*-weighted echo planar imaging sequence, and a pair of spin-echo field maps for distortion correction. Detailed MRI acquisition parameters are described in the Supplementary material.

### 4.2. Data analysis

As in the spatial working memory study, MRI data were preprocessed and analyzed using QuNex (v0.94.14; Ji et al., 2022). MRI data were preprocessed using the HCP minimal preprocessing pipelines (Glasser et al., 2013). Further analyses were performed on parcellated whole-brain data obtained by extracting the mean signal for each ROI, as identified in the HCP-MMP1.0 parcellation (Glasser et al., 2016).

All GLM task analyses were completed in two steps. The first step included assumed task regressors for the block-related response modeled separately for each incentive condition (baseline and incentive) and unassumed regressors for the transient response modeled separately for each incentive condition (baseline and incentive), each trial condition (congruent and incongruent), and for correct and incorrect responses. Assumed regressors were created by a convolution of event time series with double-gamma HRF (Friston et al., 1994a, 1998). Unassumed regressors were created by separately modeling each of the eight frames following trial onset, jointly modeling the time interval from 0 to 20 s after target stimulus onset. For the second step, the residual BOLD time series were calculated by removing the signal predicted by the linear trend and block-related regressors from the original BOLD time series. The second step included only unassumed regressors for the transient response, which were again modeled separately for each incentive condition (baseline and incentive), each trial condition (congruent and incongruent), and for correct and incorrect responses. In addition, we included a separate single unassumed regressor for all transient responses, which was scaled by the reaction times transformed into *Z*-values independently for each participant.

All sustained activity related analyses were performed using *β* estimates for the block task regressor. All analyses related to the transient response were performed on the average of the *β* estimates for frames 2 and 3 after stimulus presentation (corresponding to the period from 2.5 to 7.5 s after stimulus presentation), which were considered to be estimates of the peak trial-related transient response. Group-level statistical analyses were performed using permutation analysis (500 permutations, tail acceleration) in PALM (Winkler et al., 2014). To identify sustained and transient activation and deactivation, we performed two-tailed one-sample *t*-tests with FDR correction. We performed focused comparison between conditions by computing two-tailed paired *t*-tests with FDR correction. The significance threshold was set at *q* < .05.

To evaluate theoretical event models and construct optimized event models using autohrf, we computed a set of representative task block-related time series. To achieve this, we performed the first step as described above, however, in this case we used a single-gamma HRF (Boynton et al., 1996) to avoid any assumptions about a possible initial peak and trough at the end of the task block. We then calculated the residual BOLD signal after accounting for the intercept, linear trend, and all transient regressors and averaged the resulting time series for each brain parcel across all participants. This allowed us to extract the best estimates of task block-related response. Next, to enable unbiased optimization for a whole-brain analysis, rather than focusing on time series from regions known to be activated by the flanker task (e.g., Vanveen and Carter, 2002), we performed a hierarchical cluster analysis of the 360 time series representing each brain cluster. We used Euclidean distance as the distance measure and clustered the parcels using Ward's clustering criterion (Murtagh and Legendre, 2014). We chose Euclidean distance rather than correlation as the distance measure to emphasize (de)activation over the general shape of the time series. However, the use of correlation yielded comparable results. After clustering, we computed a representative time series by averaging the time series across all parcels in the same cluster. Finally, we computed an averaged task block response by extracting and averaging time series related to each block (4 baseline and 8 incentive blocks). Specifically, we extracted time points 1 to 32 after the start of the block, representing 80 s of the BOLD signal. These representative time series were then used to evaluate and construct the event model. For all evaluations, model fit was estimated using a weighted average *R*^2^ in which the contribution of each cluster was weighted according to the proportion of brain parcels included in the cluster.

Clustering and visualization of results were conducted using R (v4.2.1; R Core Team, 2022). Whole-brain results were visualized using Connectome Workbench (v1.5.0).

### 4.3. Results

#### 4.3.1. Construction of theoretical and data-optimized event models

**Figure 7:**
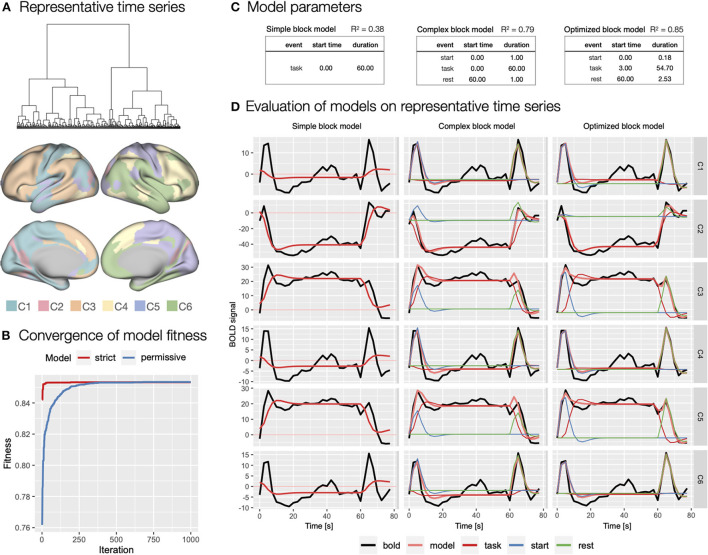
Specification and evaluation of theoretical and automatically derived event models in the flanker study. **(A)** To optimize the evaluation of the event models and facilitate comparisons, we used hierarchical cluster analysis to identify six representative time series of task block responses. The panel shows the dendrogram of the cluster analysis and the distribution of the six identified clusters across cortical parcels. **(B)** Using autohrf we explored optimization of two event models that differed by predefined constraints: a strict model closer to theoretical assumptions and a more permissive model. We ran autohrf with a population of 100 for 1000 iterations. The plot was created using the plot_fitness function. **(C)** Model parameters for the three key event models explored. A theoretical model with a single task regressor for the entire block (simple block model), a theoretical model with regressors for block start, sustained task performance, and rest (complex block model) with *a priori* event durations, and an autohrf-optimized model with event onset and duration that yielded the best model fit (optimized block model). *R*^2^ of each model is a weighted average over representative time series weighted by the number of parcels represented in each cluster. **(D)** Model fits over representative time series. BOLD time series are shown in black, time series predicted by the model in light red, while blue, red, and green show the contribution of block *start, task*, and *rest* events, respectively.

To evaluate theoretical and to generate optimized event models with autohrf, we first created representative task block related time series. Based on hierarchical cluster analysis, we identified six clusters of functional brain parcels (Figure 7A). We chose the six-cluster solution because it ensured a range of different (de)activation patterns. Visual inspection revealed that solutions with a larger number of clusters did not yield substantially different activity patterns. The representative time series can be described as indicating sustained activation (C3 - 89 parcels, C5 - 78 parcels; Figure 7A), sustained deactivation (C2 - 20 parcels), and transient activation at onset and at rest (C1 - 52 parcels, C4 - 54 parcels, and C6 - 67 parcels).

We first evaluated a theoretical event model with a single sustained *task* regressor spanning the length of the block (simple block model; Figure 7C) modeled with double-gamma HRF, possibly a most commonly used approach in block design task analysis. As indicated by the weighted *R*^2^ = 0.38, *BIC* = 216, and visual inspection of the model fit (Figure 7D), the model performed poorly, mostly due to the inability to account for transient responses at the beginning and end of the task block.

Based on the representative time series, we next evaluated a theoretical model that included separate regressors for *start* of the task block and *rest* periods (complex block model; Figure 7C). Because we had no specific basis for determining the duration of these events, we set each to an arbitrary duration of 1 s. This model performed significantly better with *R*^2^ = 0.79, *BIC* = 189. Visual inspection confirmed that the model successfully captured the transient responses at the beginning and end of the block (Figure 7D). Smaller *BIC* indicated that the improved fit was not on the account of overfitting due to additional predictors in the event model.

Finally, we used autohrf to create optimized event models based on empirical data. We ran autohrf with two sets of constraints. The first set was a more conservative set of constraints. We allowed the *start* event to vary between 0 and 2 s after block onset with a minimum duration of 0.1 s, *task* was set to occur between 0 and 60 s after block onset with a minimum duration of 55 s, while *rest* was set to occur between 60 and 65 s with a minimum duration of 0.1 s. The second set of constraints was more permissive in that the *start* event was allowed to end up to 5 s into the block, *task* regressor could end 5 s after the end of the block with a minimum duration of 50 s, and *rest* could begin already 5 s before the block ended.

Encouragingly, both sets of constraints successfully converged (Figure 7B) and resulted in very similar optimal event specifications, based on which we constructed the optimized block model (Figure 7C). The only difference was in the start time and duration of the *start* event, which were 0.06 and 0.10 s, respectively, for the strict event model and 0.00 and 0.18 s, respectively, for the permissive model. Evaluation of the optimized model showed slightly better performance with *R*^2^ = 0.85, *BIC* = 174, and visual inspection confirmed that the difference in event specification better captures the BOLD response at the beginning and end of the block (Figure 7D). Interestingly, the optimized start of the *task* regressor was at the average time of the start of the first trial in the block (mean of 2.5 and 5 s used), while the optimal end was 1.24 s after the start of the last trial in the block. We used these times in constructing new event specifications for the first step of the GLM analyses.

#### 4.3.2. Results based on theoretical and data-optimized event models

After constructing the three models, we performed GLM task analyses with each of them and compared the results. We focused on a subset of possible analyses selected to represent different types of research questions, (i) estimation of sustained and transient responses, (ii) comparison of sustained and transient responses between conditions, and (iii) evaluation of a behavioral regressor.

**Figure 8:**
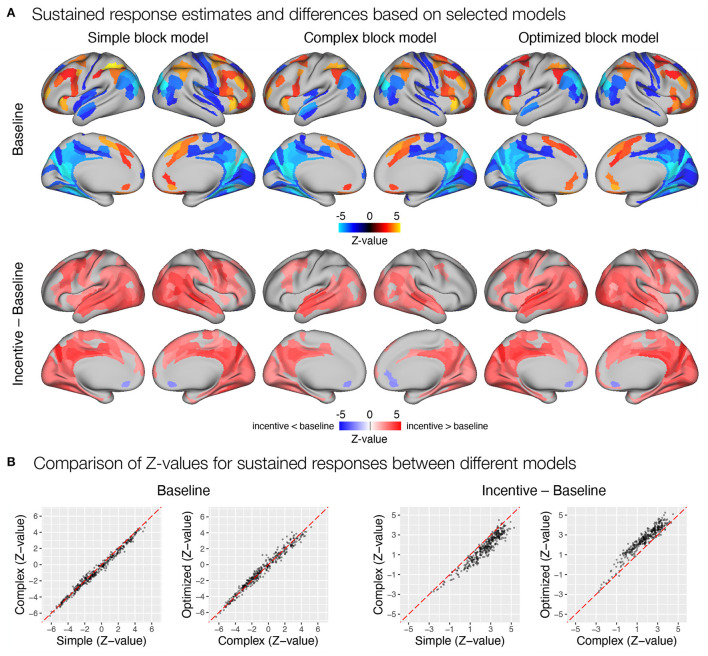
Estimation of task sustained response and incentive effects using different event models in the flanker study. **(A)** Significant sustained whole-brain activations and deactivations in baseline condition (first row) and significant differences between sustained task response in baseline and incentive conditions (second row) are shown. **(B)** A comparison of *Z*-values obtained with different models. Each point represents a *Z*-value obtained for a brain parcel using the two models compared. The red diagonal presents equal values.

First, we focused on the results related to the sustained task response, as this should be most directly affected by the differences between the three models. All three models yielded a similar activation pattern in the baseline condition (Figure 8A), with the simple block model resulting in somewhat higher positive *Z*-values, whereas the complex and optimized block models yielded similar results (Figure 8B). The slightly higher positive *Z*-values could be due to the fact that the regressor *task* must also account for transient activity at the beginning and end of the task block, which was modeled separately in the complex and optimized models. The comparison of sustained activity between baseline and incentive conditions also showed a similar pattern in all models, but the differences were more pronounced here. The complex block model resulted in substantially lower *Z*-values, which were recovered in the optimized model (Figure 8B). The latter may be due to the lack of overlap between the *task, start*, and *rest* event regressors in the optimized model, which allows *task* sustained activity to be estimated fully independently of the transient responses at the beginning and end of the task block.

**Figure 9:**
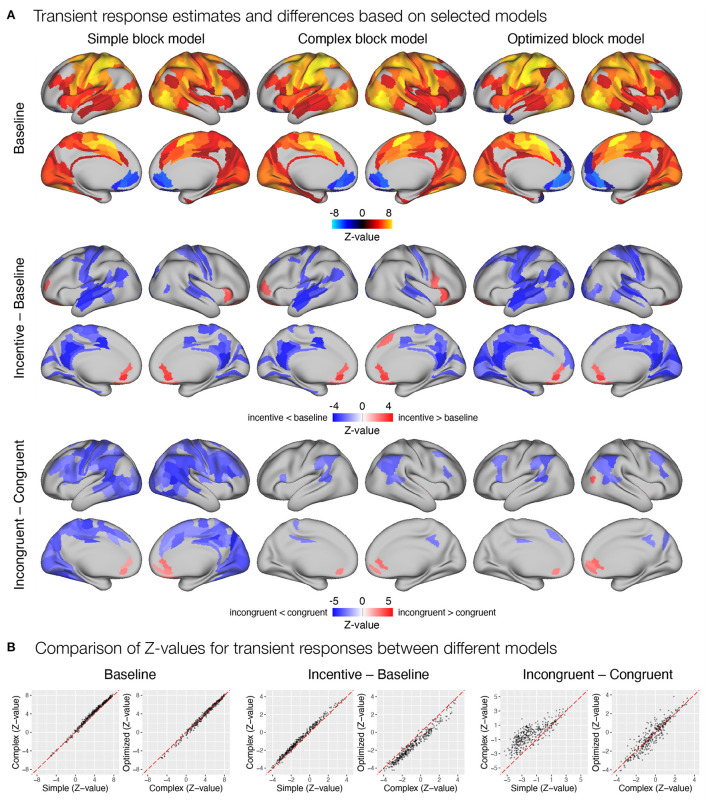
Estimation of transient task response, incentive and stimulus congruency effects using different event models in the flanker study. **(A)** Significant transient whole-brain activations and deactivations in the baseline condition (top row), significant differences in transient response between incentive and baseline conditions (middle row), and significant differences between responses to incongruent and congruent stimuli in baseline condition (bottom row) are shown. **(B)** A comparison of *Z*-values obtained with different models. Each point represents a *Z*-value obtained for a brain parcel using the two models compared. The red diagonal presents equal values.

Next, we examined how different modeling of the task block response affected estimates of trial-related transient responses. Again, all three models yielded a similar pattern of significant activations and deactivations across cortical parcels (Figure 9A). Here, the complex block model yielded slightly higher positive *Z*-values (Figure 9B), resulting in more identified significant activations in frontal and temporal cortex. The optimized block model, on the other hand, identified additional posterior parietal activations and deactivations in the medial prefrontal cortex and left temporal pole. The comparison of the significant effects of the incentive on the transient responses differed substantially between the three models (Figure 9A). Whereas the simple model resulted in a small number of decreases in responses in the incentive condition, the complex model identified a number of increases in task-relevant parcels, and the optimized model identified no significant differences. Both the *Z*-values (Figure 9B) and the examination of un-thresholded whole-brain images (Supplementary Figure S15A) revealed an overall shift from decreases in the simple model to increases in the complex and optimized models. It appears that the transient responses at the beginning and end of the block captured by the *task* regressor in the simple model mask the increase in transient responses, leading to misleading results in the simple model and overestimates in the complex model. A similar observation can be drawn from the comparison of incongruent and congruent responses within the baseline block, where the simple model indicates a large number of reduced transient responses in incongruent compared to congruent trials, while the complex and optimized models again provide more comparable results (Figure 9).

**Figure 10:**
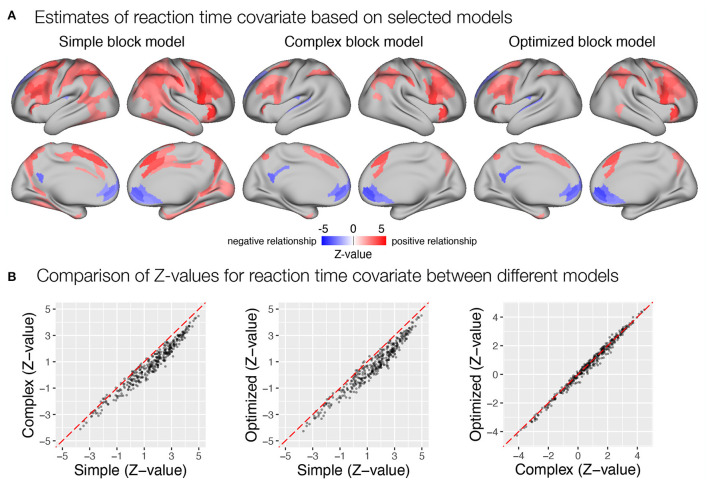
Estimation of correlation between trial-to-trial variability in transient response and reaction times in the flanker study. **(A)** Whole-brain maps of significant *β* estimates reflecting covariability between trial-to-trial transient responses and reaction times are shown. **(B)** A comparison of *Z*-values obtained with different models. Each point represents a *Z*-value obtained for a brain parcel using the two models compared. The red diagonal presents equal values.

Last, we examined the extent to which the different models captured the correlation between transient responses and trial-to-trial reaction times. Similar to previous results, the simple block model yielded a larger number of parcels identified as significantly correlated with reaction times than the complex and optimized block models (Figure 10). It should be noted here that the reaction time covariate was computed across all trials in all task blocks, which differed in mean reaction times, as noted earlier. If there were differences in sustained responses between conditions that were not captured by the sustained regressor, they would affect the estimates of the transient response. Transient responses would be overestimated if sustained activity was underestimated and vice versa. This appears to have been the case when the simple model was used. A slight overestimation of sustained responses in the incentive condition and an underestimation in the baseline condition would result in an overestimation of the magnitude of transient responses in the baseline condition and an underestimation in the incentive condition, which would be reflected in an increase in the positive correlation with reaction times. This pattern is also consistent with the previously observed paradoxical reduction in transient responses in the incentive condition compared with the baseline condition when the simple model is used (Figure 9A), as well as the observation of a slightly higher increase in estimates of sustained activity in the incentive condition compared with the baseline condition (Figure 8A).

Jointly, the review of results obtained with the three models indicate a high probability of invalid results obtained with the simple block model and possible additional improvements in validity when using the autohrf-optimized model.

## 5. Discussion

The autohrf package provides a unique and novel approach to event model construction for the GLM analysis of task-related fMRI data. Although GLM is a powerful tool for decomposing BOLD signal and estimating individual responses to neural events, it is limited by the relatively arbitrary specification of the event model used to describe the BOLD signal (Luo and Nichols, 2003; Lindquist, 2008; Loh et al., 2008; Poldrack et al., 2011; Pernet, 2014). Instead of defining event models based on predefined assumptions, we present an approach to constructing the event model based on the measured BOLD signal. In addition, supporting functions provide informative evaluation and visualization of the constructed models. While the autohrf package does not replace the necessary domain-specific knowledge about the cognitive processes under study and how they are reflected in the BOLD signal, it does provide the researcher with additional information to make an informed decision when designing event models for the GLM analysis.

We have demonstrated the utility of the package on two example datasets and a data simulation. The diverse behavioral tasks and study designs used in both datasets suggest broad applicability of the autohrf package. Nevertheless, the focus of our analysis was to evaluate the performance and reliability of the automatically derived models compared to theoretically defined models. We were interested in the fit of the models, the changes in effect sizes of the resulting response estimates, and any qualitative differences in the observed patterns of results. Though the optimized models have not always resulted in higher effect sizes, the qualitative review of the specific patterns of results indicates an improvement in the validity of the results. It needs to be noted that the use of autohrf to evaluate theoretical event models also led to better theoretically defined event models that yielded results with higher validity. In any case, we have to appreciate that even small changes to the event model resulted in considerable qualitative differences in the final results. These results suggest that the model choice is an impactful step in task-related fMRI analysis. Still, the reliability of modeling the HRF and event timing used in task-related fMRI studies is rarely evaluated (Lindquist, 2008).

Based on the spatial working memory study, we presented an example procedure for selecting the most appropriate event model using the autohrf package. First, our results showed that the choice of the number of task events substantially affects the obtained response estimates. Too few events are sometimes unable to account for enough variability in the BOLD response, while too many events can lead to inappropriate GLM estimates. Second, as expected, the automatically derived event timing parameters provided a better fit to the actual BOLD response compared with the theoretically defined models. However, the face validity of the GLM estimates appeared to decrease with the relaxation of the predefined constraints passed to autohrf. During visual inspection, this was evident in the form of over- or underestimates and unexpected shifts in the timing of events. This may suggest that the theoretical assumptions about brain activity still provide necessary information, whereas autohrf can be used to fine-tune event specification. However, it is also possible that autohrf reveals incorrect assumptions about cognitive processes and associated neural activity in our task designs and could provide an analytic tool for examining the detailed properties of the components of the BOLD task response. Finally, performing statistical analyses of response estimates obtained with automatically derived compared to theoretical models revealed substantial qualitative differences in activity patterns and task differences. In some cases and brain regions, theoretical models led to higher effect sizes, and automatically derived ones in others. Because of this variability, we were unable to determine which type of model produced unambiguously better results. This highlights the possibility that it is not optimal to use a single event model for the whole brain because of differences in the timing of component cognitive processes and associated neural activity in different brain systems and regions. We see two possible ways to address this issue. One is to extend autohrf to optimize event models independently for different brain systems. Another is to develop deconvolutional approaches that do not depend on explicit specification of neural events.

Additional validation of the models optimized with autohrf using cross-validation and data simulation also provided valuable information. The autohrf models optimized on the first session performed better than the theoretical model in subsequent sessions, and the models optimized on different sessions were well matched. Both results indicate that the autohrf results are stable and reflect the event structure of the data rather than capitalize on noise. Simulation of data from a working memory task also showed that autohrf can reliably reconstruct the event structure present in the signal and adapt to HRF differences. However, performance is better for events of longer duration. The *β* estimates of activity capture the true variability very well, but do not provide a perfect decomposition as they can still be affected by the preceding and following events.

The flanker study provided another example of the usefulness of the autohrf package in both evaluating theoretical and constructing optimized empirical models. The creation of representative time series allowed for more efficient and interpretable investigation and review of various event models. Evaluation of the initial model encouraged the development of a better theoretical model of block task response, while further optimization revealed additional valuable features of the BOLD response. Comparison of the constructed models again revealed the extent to which modifications of event models lead to important qualitative differences in results beyond changes in effect sizes. The observed changes are not always obvious, as an improvement in the modeling of one event can lead to considerable changes in other estimates. In the example of the flanker study, a change in the modeling of a sustained regressor led to appreciable differences in transient responses, including trial-to-trial behavioral correlations.

The two example studies were selected to illustrate the use cases for evaluating and optimizing event models with autohrf. They enabled the identification of a significant impact of event modeling decisions on final results and provided valuable insights into slow-event and mixed state-item designs commonly used in functional neuroimaging. We primarily focused on demonstrating different ways of using autohrf and the differences in the results obtained. While we have described the main observations in the pattern of results, we have not explored in depth the specific differences and underlying mechanisms. Although additional insights could be gained by testing different explanations for the observed results, this was not the main focus of the paper. We hope that the observations presented will stimulate further investigation by other researchers in the field and promote better understanding and appreciation of the task-related GLM decisions.

### 5.1. Limitations

One of the current limitations of the package is the increase in processing time required for automatic parameter search with the increase in the number of ROIs, population size, and iterations. On a 2019 MacBook Pro, a single iteration for a study with 37 participants, and a population size of 100, required about 10 s when run across 36 ROIs, and 100 s when run across 180 ROIs. An automated parameter search for a model with the default 100 iterations takes about 20 min for 36 ROIs and about 3 h for 180 ROIs. These times can be significantly reduced when using a modern desktop computer or a dedicated compute server. The processing time required remains manageable when working with most commonly used parcellations. For example, we have successfully applied the autohrf package to the data consisting of 360 ROIs defined in the HCP-MMP1.0 parcellation (Glasser et al., 2016). Beyond that the duration of the automated search increases rapidly, making voxel- or grayordinate-based analyses infeasible in the current version of the package.

When using the autohrf function with model constraints of varying complexity as inputs, one must acknowledge the fact that more complex models are more flexible and will always fit the data better. Therefore, to avoid overfitting of the model it is best to use techniques developed in machine learning. The most common approach to address overfitting is cross-validation. In cross-validation, a subset of data is used to train/fit the model. After training is complete, the quality of the model is evaluated using unseen test data. If the model is overfitted to the training data, it does not generalize well to independent data and receives a lower evaluation score. We presented an example of a hold-out cross-validation on the spatial working memory study, where we used the fMRI data from the first recording session as an input to the autohrf function for the purpose of automated parameter search. We then tested the obtained models using the evaluate_model function on the fMRI data from the remaining second and third sessions. With this procedure, model evaluation was performed on the data that were not used for automated parameter search. It should be noted that the training and testing datasets should be carefully selected to avoid introducing systematic factors that could affect the generalizability of the model. For example, conducting cross-validation across multiple sessions could be problematic if there are extended periods of time between sessions due to potential developmental changes (e.g., Herting et al., 2018) or disease progression (e.g., Han et al., 2022). Since data acquisition in neuroimaging is often costly and datasets are small, cross-validation is not always feasible. In such cases, the user should consider the *BIC* measure reported by the evaluate_model, which accounts for model complexity. Note that appropriate cross-validation is more robust and reliable than *BIC*, so cross-validation is preferable when sufficient data is available.

### 5.2. Future developments

We consider autohrf in its current form a useful proof-of-concept that we intend to develop further in terms of both additional functionality and analytical capabilities. Features that are already in the pipeline but did not make it into the initial release include the ability to perform automatic search for model parameters per participant and per ROI basis. Because studies show considerable variability in hemodynamic responses between brain areas and participants (Aguirre et al., 1998; Miezin et al., 2000; Handwerker et al., 2004; Badillo et al., 2013), we aim to further develop a separate estimation of model parameters for individual ROIs and participants that could account for this variability in BOLD response and thus provide more reliable estimates of brain activity. In addition, such a tool could also be used to study individual differences in BOLD responses as well as the hemodynamic properties of different brain systems.

To extend the utility of the autohrf package, we plan to incorporate other types of BOLD response modeling, such as unassumed modeling. Additionally, we plan to integrate the autohrf package into QuNex (Ji et al., 2022), which would allow an easier use of the models obtained with autohrf in large-scale fMRI data analysis.

As a part of an additional evaluation analysis, we plan to investigate how different parameters in genetic algorithms affect the models obtained with autohrf. The default values for the number of iterations, population size, and mutation rate in genetic algorithms were set based on a series of experiments using our own data with a goal to provide the convergence of models in a reasonable time frame. Since the optimal values for these parameters are study-dependent, some users may need to optimize them through their own experiments. We plan to analyze the effects of these parameters on model convergence and results in more detail in the future. This should allow users to set better values for their studies more efficiently.

### 5.3. Conclusion

The exploration of automatic optimization of event models based on empirical data suggests that autohrf can be used to check and validate theoretical models, to develop optimized models that result in a better fit to observed BOLD responses, and to explore the event structure underlying task BOLD responses. Using autohrf with reasonably defined constraints may lead to more valid and reliable estimates of brain activity and higher statistical power. Thus, autohrf can serve as a powerful tool that can be used in an easy and efficient manner in combination with other analytic tools to support and guide large-scale GLM analysis of task-related fMRI data. Furthermore, we show that the automatic parameter search enabled by autohrf can provide unique information about the properties of fMRI signals that challenge existing assumptions and open a new analytical approach in functional neuroimaging.

## Data availability statement

The source code of autohrf can be found in the GitHub repository at https://github.com/demsarjure/autohrf. The package is also available on CRAN, so users can install it via R's install.packages function. In the package, users will find condensed datasets of the two studies presented in the paper, the spatial working memory study and the flanker study. In addition, we have also included example analysis steps using the autohrf package based on the data from the two studies that can be used to test and reproduce the results reported in the paper. The datasets and scripts used for the evaluation analysis based on the spatial working memory study, spatial working memory simulation, and the flanker study are also available in the OSF repository at https://osf.io/amhb2/.

## Ethics statement

The studies involving human participants were reviewed and approved by the Ethics Committee of the Faculty of Arts, University of Ljubljana, Ljubljana, Slovenia, while the spatial working memory study was additionally approved by the National Medical Ethics Committee, Ministry of Health of the Republic of Slovenia, Ljubljana, Slovenia. The participants provided their written informed consent to participate in this study.

## Author contributions

NP, JD, AA, and GR: conceptualization. JD and GR: methodology and software. NP and GR: investigation and formal analysis. NP, JD, and GR: writing—original draft, writing—review, and editing. AA and GR: resources, supervision, and funding acquisition. All authors contributed to the article and approved the submitted version.

## Funding

This work was supported by the Slovenian Research Agency (Young Researcher program and the research Grant Nos. J3-9264, P3-0338, and P5-0110) and the CIMA General Charitable Trust.

## Conflict of interest

JD consults for Manifest Technologies. AA and GR consult for and hold equity in Neumora Therapeutics and Manifest Technologies. The remaining author declares that the research was conducted in the absence of any commercial or financial relationships that could be construed as a potential conflict of interest.

## Publisher's note

All claims expressed in this article are solely those of the authors and do not necessarily represent those of their affiliated organizations, or those of the publisher, the editors and the reviewers. Any product that may be evaluated in this article, or claim that may be made by its manufacturer, is not guaranteed or endorsed by the publisher.
