# Corrigendum: autohrf-an R package for generating data-informed event models for general linear modeling of task-based fMRI data

**DOI:** 10.3389/fnimg.2023.1158159

**Published:** 2023-02-21

**Authors:** Nina Purg, Jure Demšar, Alan Anticevic, Grega Repovš

**Affiliations:** ^1^Department of Psychology, Faculty of Arts, University of Ljubljana, Ljubljana, Slovenia; ^2^Faculty of Computer and Information Science, University of Ljubljana, Ljubljana, Slovenia; ^3^Department of Psychiatry, Yale University School of Medicine, New Haven, CT, United States; ^4^Department of Psychology, Yale University School of Medicine, New Haven, CT, United States

**Keywords:** fMRI, GLM, assumed modeling, task-related activity, autohrf, R

In the published article, there was an error in the author list, and author Alan Anticevic was erroneously excluded. The corrected author list appears below.

Nina Purg^1*^†, Jure Demšar^1,2^†, Alan Anticevic^3,4^ and Grega Repovš^1^

^1^Department of Psychology, Faculty of Arts, University of Ljubljana, Ljubljana, Slovenia

^2^Faculty of Computer and Information Science, University of Ljubljana, Ljubljana, Slovenia

^3^Department of Psychiatry, Yale University School of Medicine, New Haven, CT, United States

^4^Department of Psychology, Yale University School of Medicine, New Haven, CT, United States

In the published article, there was an error. Alan Anticevic was erroneously excluded from the Author Contributions statement. The corrected Author Contributions statement is below.

NP, JD, AA, and GR: conceptualization. JD and GR: methodology and software. NP and GR: investigation and formal analysis. NP, JD, and GR: writing—original draft, writing—review, and editing. AA and GR: resources, supervision, and funding acquisition. All authors contributed to the article and approved the submitted version.

In the published article, there was an error. Alan Anticevic was erroneously excluded from the Conflict of Interest statement. The corrected Conflict of Interest statement is below.

JD consults for Manifest Technologies. AA and GR consult for and hold equity in Neumora Therapeutics and Manifest Technologies.

The remaining author declares that the research was conducted in the absence of any commercial or financial relationships that could be construed as a potential conflict of interest.

The authors apologize for this error and state that this does not change the scientific conclusions of the article in any way. The original article has been updated.

