# Supplementary material for: autohrf-an R package for generating data-informed event models for general linear modeling of task-based fMRI data
Source: Front Neuroimaging. 2022 Dec 5;1:983324. doi: 10.3389/fnimg.2022.983324 (PMC10406192; doi:10.3389/fnimg.2022.983324)
Supplement: Supplementary file 1 [file Data_Sheet_1.pdf]

Supplementary Material

1 SUPPLEMENTARY METHODS

1.1 Calculation of the Boynton and SPM hemodynamic response functions

The `autohrf` package currently offers the use of two assumed hemodynamic response functions (HRF), Boynton (Boynton et al., 1996) and SPM (Friston et al., 1994, 1998). Both, in the case of Boynton HRF and in the case of SPM HRF, HRF is represented as a vector $\vec{h}$ of length n, where $n = \lceil t/tr \rceil$. The `tr` parameter defines MRI's repetition time in seconds and the `t` parameter defines the duration of the generated HRF in seconds. The Boynton's method generates HRF as:

$$\vec{y} = [y_1, ..., y_n],$$
$$y_i = (i-1) \cdot tr; i \in 1, \ldots, n,$$
$$\vec{r} = \frac{(\vec{y} - \delta)}{\tau},$$
$$\vec{h} = r^{\alpha} e^{r},$$
$$h_i = \begin{cases} 0, & \text{if } h_i < \delta \\ h_i, & \text{otherwise} \end{cases},$$
$$\vec{h} = \frac{\vec{h}}{\alpha^{\alpha} e^{-\alpha}}.$$

The δ, τ and α parameters of the Boynton's HRF can be fine-tuned in the $p_{boynton}$ parameter. $p_{boynton} = [\delta, \tau, \alpha]$, by default $p_{boynton} = [2.25, 1.25, 2]$.

If SPM HRF is used then the vector h is generated as:

$$\Delta t = tr/t,$$
$$u_i = (i-1)t - \frac{p_6}{\Delta t}; i \in 1, \ldots, n,$$
$$h_i = \frac{\gamma(u_i \frac{\Delta t}{p_3}, \frac{p_1}{p_3}) - \gamma(u_i \frac{\Delta t}{p_4}, \frac{p_2}{p_4})}{p_5},$$
$$h_i = \frac{h_i}{\max(\vec{h})},$$

where γ is the density function of the gamma distribution and $p_1, p_2, p_3, p_4, p_5, p_6$ are the parameters of the SPM HRF. These parameters are set through the $p_{spm} = [p_1, p_2, p_3, p_4, p_5, p_6]$ parameter, which by default is $p_{spm} = [6, 16, 1, 1, 6, 0]$.

1.2 General linear modeling of the fMRI signal

In the `autohrf` package, the measured fMRI signal is modeled using general linear modeling (GLM). First, the constructed HRF is convolved with the neural time series generated from the event descriptions:

$$c_i(t) = n_i(t) \star \text{HRF}(t),$$
$$n_i(t) = \begin{cases} 1, & \text{if } e_{i,start} \leq t \leq e_{i,end} \\ 0, & \text{otherwise} \end{cases},$$

where $c_i(t)$ is the predicted BOLD time course for the event i at time t, $n_i(t)$ denotes the value of neural activity for event i at time t, while $e_{i,start}$ and $e_{i,end}$ denote the start and end times for event i.

Next, to evaluate the quality of the predefined event model a linear model is fitted:

$$y_j = \beta_0 + \beta_1 c_1 + \beta_2 c_2 + \ldots + \beta_n c_n + \epsilon,$$

where y_j is the BOLD signal for ROI j, β_0 is the intercept, $\beta_1 \ldots \beta_n$ are the regression coefficients of the linear model, $c_1 \ldots c_n$ are the event predictors, and ϵ is the residual.

1.3 Calculation of the mutation event in genetic algorithms

Genetic algorithms used in the automated parameter search include mutation events, which mimic the natural phenomenon where genes in the DNA of species can randomly mutate and change as a result. In the case of the `autohrf` function, mutation slightly changes the value of the onset or offset of an event. For each event and start/end time, there is a probability, equal to the `mutation_rate` that a mutation event will be triggered. With mutations, we ensure that genetic algorithms not only search for the best solution within the randomly generated initial population, but also explore new solutions that may be better than those in the existing population. If the mutation event is triggered for the onset of event i, then the new onset of that event becomes:

$$e_{i,start} = e_{i,start} + \mathcal{U}(-d\, m_f, d\, m_f),$$

where d is the duration of event i, calculated as $e_{i,end} - e_{i,start}$, m_f is the value of the `mutation_factor` parameter, and $\mathcal{U}$ is the uniformly distributed random number generator function.

2 THE SPATIAL WORKING MEMORY STUDY

2.1 MRI data acquisition and preprocessing

MRI data were collected with Philips Achieva 3.0T TX scanner. T1- and T2-weighted structural images were acquired for each participant (T1 and T2: field of view = 224 × 235 mm, 236 sagittal slices, matrix = 320 × 336, voxel size = 0.7 × 0.7 × 0.7 mm; T1: TE = 5.8 ms, TR = 12 ms, flip angle = 8°; T2: TE = 394 ms, TR = 2500 ms, flip angle = 90°). Brain activity was measured using BOLD images with T2*-weighted echo-planar imaging sequence (2-6 BOLD images, field of view = 240 × 240 mm, 56 axial slices, voxel size = 2.5 × 2.5 × 2.5 mm, matrix = 96 × 95, TR = 1000 ms, TE = 48 ms, flip angle = 62°, MultiBand SENSE factor 8, 698 frames).

MRI data were preprocessed and analyzed using Quantitative Neuroimaging Environment & Toolbox (QuNex; Ji et al., 2022). Image preprocessing was performed using the Human Connectome Project (HCP) minimal preprocessing pipelines (Glasser et al., 2013). The structural images were corrected for magnetic field distortions and registered to the MNI atlas, brain tissue was segmented into white and gray matter, and the cortical surface was reconstructed. Functional BOLD images were slice-time aligned, corrected for spatial distortions, motion corrected, registered to the MNI atlas, and the BOLD signal was mapped to a common surface volume (CIFTI) representation.

2.2 Simulated working memory fMRI data

To further validate `autohrf` package, we analyzed the performance of the package on simulated datasets. Specifically, we simulated BOLD responses during a working memory task using four different event models, three different HRFs, and five different noise levels, resulting in 60 different simulated datasets. We used the datasets to evaluate (i) the ability of `autohrf` to identify the original response, (ii) the robustness of `autohrf` to different noise levels, (iii) the ability to adjust identified event models to differences in the HRF, and (iv) the advantage of `autohrf`-optimized event models in estimating neural activity.

2.2.1 Generation of synthetic BOLD time series

Simulated event models: Synthetic BOLD signal was computed by convolving event time series with a double-gamma HRF and adding Gaussian noise. We used event times series specified by four different event models (Figure S1). Each model included *encoding*, *delay*, and *response* events. Two models were chosen based on theoretical assumptions of cognitive processes during a working memory task trial (Models A and B; Figure S1), while two models were estimated empirically using `autohrf` based on the fMRI data collected during the spatial working memory task (Models C and D; Figure S1). Besides the timing information, the models also included the reference activation values for each of the events. The reference values were chosen to recreate BOLD response typically observed in ROIs activated by the task.

Simulated HRF: We generated the simulated data using three different HRFs that differed in the time-to-peak, namely, 4, 5, and 6 s. The times were chosen to include the default value typically used with double-gamma HRF (5 s) and roughly cover the range of empirically measured HRF (Aguirre et al., 1998; Miezin et al., 2000; Handwerker et al., 2004). The exact parameters used are presented in Table S1.

Table S1. HRF parameters used in BOLD signal simulation

HRF	p1	p2	p3	p4	p5	p6	p7
HRF 1	5	15	1	1	6	0	32
HRF 2	6	16	1	1	6	0	32
HRF 3	7	17	1	1	6	0	32

Simulated noise levels: We simulated noise by adding random Gaussian noise to the generated time series. To simulate different levels of noise, we manipulated the SD of the Gaussian distribution from which the random values were sampled. We used the following five SDs: 0, 0.067, 0.1, 0.15, and 0.225. The values were chosen to systematically cover a wider range of possible noise levels and were informed based on visual comparison with the empirical BOLD signal.

Simulated ROIs: For each of the 60 unique combination of an event model, HRF time-to-peak and noise level we simulated BOLD time series for 10 ROIs that differed in the simulated response magnitude to individual events in the model. In that way, we ensured enough variability in BOLD responses which is used

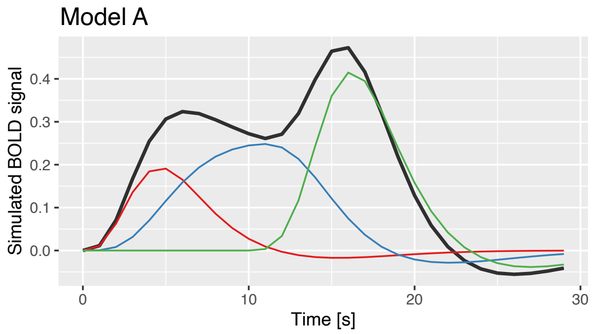

event	time	duration	value
encoding	0.00	0.15	7.00
delay	0.15	9.85	0.25
response	10.00	3.00	0.80

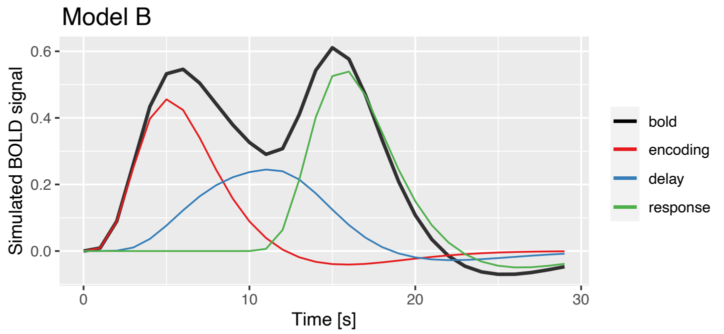

event	time	duration	value
encoding	0.00	1.00	2.50
delay	1.00	9.00	0.25
response	10.00	2.00	1.50

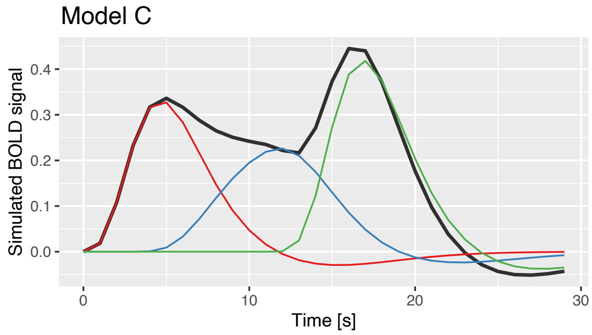

event	time	duration	value
encoding	0.08	0.07	12.00
delay	3.14	6.86	0.25
response	11.50	1.50	1.50

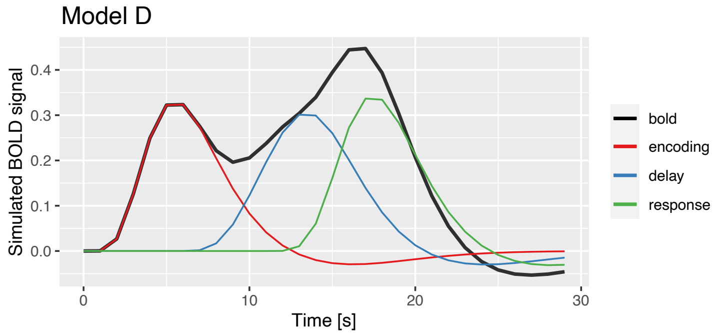

event	time	duration	value
encoding	0.90	0.10	12.00
delay	6.00	5.00	0.40
response	11.60	2.40	0.80

Figure S1. Event models used in working memory fMRI data simulation. Four models were used with *encoding*, *delay*, and *response* events, which differed in the event onset and duration.

by `autohrf` to obtain reliable estimates of event parameters. The 10 ROIs were created by multiplying the event reference values for each model with a set of activation weights (Table S2). The activation weights were chosen to mimic the BOLD responses observed in the task. The resulting time series for all ROIs across all models are shown in Figure S2.

Table S2. Simulated response magnitudes to specific events for individual ROIs

roi	encoding	delay	response
ROI 1	1.00	1.00	1.00
ROI 2	0.70	1.00	1.00
ROI 3	1.00	0.70	1.00
ROI 4	1.00	1.00	1.00
ROI 5	0.70	0.70	1.00
ROI 6	0.70	0.30	1.00
ROI 7	1.00	0.00	1.00
ROI 8	1.00	-0.50	1.00
ROI 9	0.50	-1.00	1.00
ROI 10	0.20	-1.20	1.00

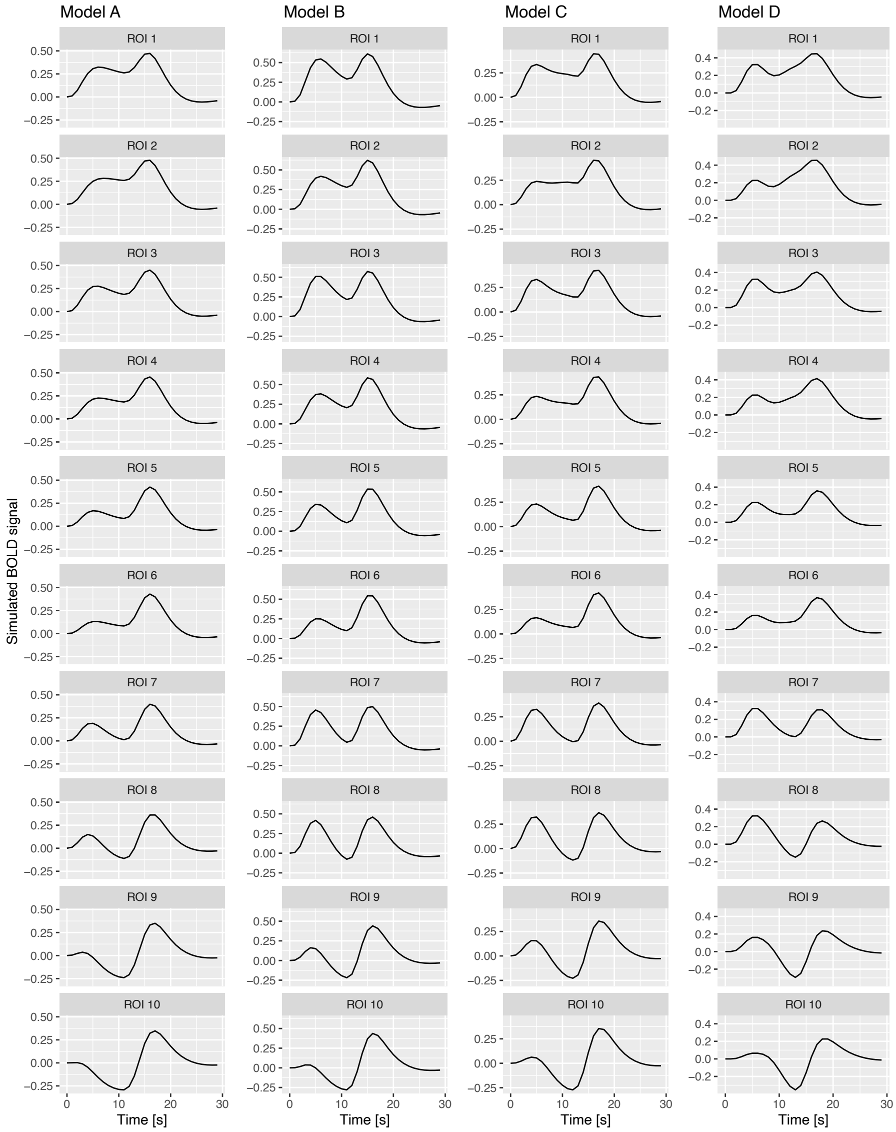

Figure S2. Simulated BOLD responses for individual ROIs. For each of four event models, 10 ROIs were prepared with differing response magnitude.

2.2.2 Running automated parameter search based on simulated BOLD signal

Based on each individual BOLD signal simulation we ran the automated parameter search across 10 ROIs using the `autohrf` function. Specifically, we ran two models, one with strict constraints and another with more permissive constraints. Constraints were carefully chosen to include event timings across different event models used in data simulation. Detailed constraints for both models can be found in Table S3. We used a population size of 100 and 500 iterations.

Table S3. Model constraints used in the automated parameter search

Strict model

event	start time	end time	min duration	max duration
encoding	0.00	1.00	0.05	1.00
delay	0.00	10.00	5.00	10.00
response	10.00	13.00	1.00	3.00

Permissive model

event	start time	end time	min duration	max duration
encoding	0.00	2.00	0.05	2.00
delay	0.00	11.00	5.00	11.00
response	9.00	14.00	1.00	5.00

2.2.3 Evaluation of reconstructed event timing

To evaluate the robustnest of `autohrf` to different noise levels we computed an overlap in event timing across noise levels, separately for each simulated event model and HRF timing. Specifically, the overlap was computed as the percentage of time covered by the automatically obtained event parameter across all noise levels, divided by the entire time range that included this event in at least one of five noise simulations.

To quantitatively test the ability of `autohrf` to obtain valid event predictors we calculated the overlap between automatically estimated parameters and simulated event timing that was used to generate the synthetic BOLD signal. The overlap was computed as a percentage of time that was covered by the estimated parameter in relation to the time range covered by the simulated event, separately for each event model, HRF timing and noise level.

Finally, we checked the sensitivity of `autohrf` to different HRF time-to-peak. We were interested to what extent the estimated parameters overlap with the simulated event timing despite variable HRF and whether the `autohrf` is able to adjust for this variability. Specifically, we again computed a percentage of time that was covered by the estimated parameter in relation to the time range covered by the simulated event, separately for each event model, HRF timing and noise level.

2.2.4 Evaluation of estimated β values

The final series of tests involved evaluating the impact of `autohrf` optimization of event models on the quality of β estimates of neural activity compared to using theoretically motivated event models. For this part of the evaluation, we generated a new series of 60 simulated datasets with the same parameters as for the original simulated data, but for a new set of ROI. In this case, we simulated a total of 216 ROI, covering a permutation of 6 different activation levels for each event, *encoding*, *delay*, and *response*. Activations

were again generated by multiplying a set of weights by the event reference values for each of the four simulated models. The list of weights for each event is provided in Table S4.

Table S4. The set of activation weights for each event

event	activation weights					
encoding	-0.25	0.00	0.25	0.50	0.75	1.00
delay	-1.00	-0.50	0.00	0.50	0.75	1.00
response	-0.25	0.00	0.25	0.50	0.75	1.00

In each dataset, we first calculated the simulated BOLD responses for each ROI and then used GLM to estimate the activity associated with each of the events using four event models, two theoretical models that matched Models A and B used to generate the simulated data and two models that were optimized with `autohrf` using the original simulated data with strict and permissive constraints. For each simulated ROI, the original activation values used to generate the simulated BOLD time series and β estimates obtained with the four models were recorded.

The wide range of combinations across simulated ROIs allowed us to (i) test the recovery of the simulated activations and (ii) assess the extent to which the β estimates were affected by the variability of preceding and/or subsequent events, e.g., the extent to which the amplitudes of *enconding* and *response* activity affected the estimated β value for *delay* activity.

We examined success in recovering simulated activations by calculating the Pearson's correlation between simulated activities and β estimates. We calculated the correlations across all 216 simulated ROIs for each event, each estimation model, and each dataset separately. To create a summary figure (Figure 6E), we divided the results into those for matching theoretical models (i.e., when the theoretical estimation event model matched the event model used to generate the data), non-matching theoretical models (i.e., when the theoretical event model did not match the event model used to generate the data), `autohrf`-optimized models using strict constraints, and `autohrf`-optimized models using permissive constraints. For each of these situations, we then calculated the average and 95% confidence interval using fast bootstrap across noise levels.

Comparison of correlations obtained with matching theoretical models and optimized models at HRF time-to-peak 5 s shows how well the empirically derived optimized models perform compared to the ideal matching models. The same comparison, but with HRF time-to-peak 4 s and 6 s shows the extent to which optimized models can adapt to non-matching HRF. Comparison of the optimized models with the non-matching theoretical models shows the extent to which the optimized models can outperform the theoretical models when the timing assumed by the theoretical model does not match the actual processes that generate the BOLD response.

Finally, to evaluate the event decomposition, we calculated the range of β estimates for each estimation model for each dataset and event separately across all ROIs where the simulated activity was stable using the formula:

$$\beta_{span} = abs(max(\beta) - min(\beta))$$

β_{span} thus indicated what the maximum span of values was in cases where the β estimate was expected to be stable. In these cases, any differences between the estimated β values can be attributed either to noise

or to the influence of the changes in the activation level of the preceding and/or subsequent events on the estimate.

We calculated mean spans across all 6 stable levels for each event separately for each combination of models used to simulate the data, HRFs, noise levels, and models used to calculate the β estimates. The summary results shown in Figure 6F were calculated in the same manner as for Figure 6E.

3 THE FLANKER STUDY

3.1 MRI data acquisition and preprocessing

MRI data were acquired with Philips Achieva 3.0T TX scanner. T1-weighted and T2-weighted high-resolution, whole-brain anatomical scans were acquired (both: 236 sagittal slices, matrix = 336×336, voxel size = $0.7 \times 0.7 \times 0.7$ mm; T1: TE = 5.7 ms, TR = 12 ms, flip angle = $8°$; T2: TE = 414 ms, TR = 2.5 s, flip angle = $90°$). Whole-brain functional volumes (BOLD) were acquired with a T2*-weighted echo planar imaging sequence (48 axial slices, voxel size = $3 \times 3 \times 3$ mm, matrix = 80×80, TR = 2.5 s, TE = 27 ms, flip angle = $90°$, SENSE factor 2) in three bold runs (146 frames each). In addition, to support distortion correction of both structural and functional images, two spin-echo images (48 axial slices, voxel size = $3 \times 3 \times 3$ mm, matrix = 80×80, TR = 2.5 s, TE = 27 ms, flip angle = $90°$, SENSE factor 2) were acquired with opposite frequency readout directions (anterior-to-posterior and posterior-to-anterior).

As in the spatial working memory study, MRI data were preprocessed and analyzed using QuNex (Ji et al., 2022). Images were preprocessed using the HCP minimal preprocessing pipelines (Glasser et al., 2013) and mapped to a common surface volume representation (CIFTI).

4 SUPPLEMENTARY FIGURES

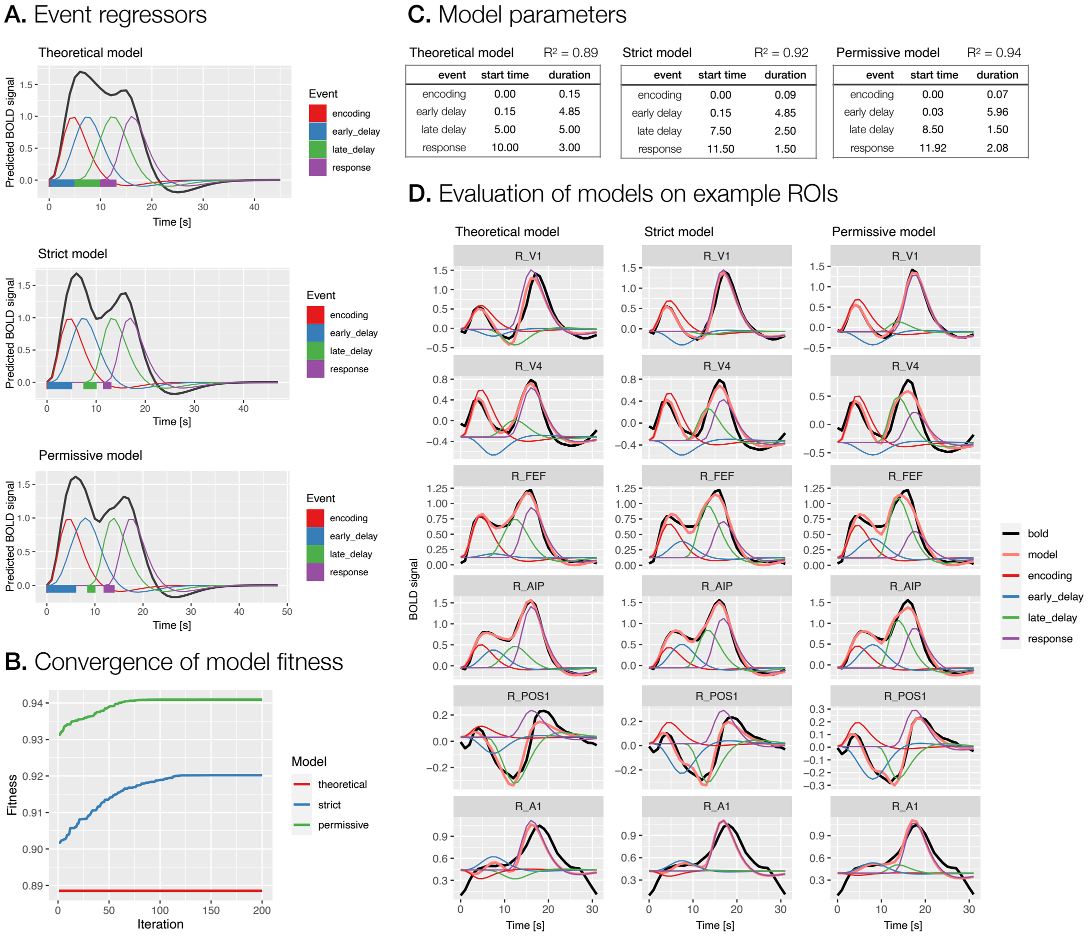

Figure S3. Specification and performance of theoretical and automatically derived event models in the spatial working memory study. Here, we compare three models differentiating between *encoding*, *early delay*, *late delay* and *response* events, a theoretically derived model based on the assumed timeline of events in the task and two automatically derived models based on empirical BOLD signal using the `autohrf` function that differed in the predefined constraints, the permissive model was allowed larger deviations from the theoretical model than the strict model. The models were fit to the activity of a predefined selection of 80 ROIs in the frontoparietal network, in addition to visual and motor-related brain areas that were assumed to show responses to the task. **A.** A visualization of task event predictors convolved with double-gamma HRF obtained using the `plot_best_models` function, where the colored lines depict individual responses to specific events and the black line shows the summation of these responses in the BOLD signal. Rectangles at the bottom visualize the onset and duration for each of the events in the model. **B.** The convergence of the model fitness in the automated parameter search based on the population of 100 and 200 iterations. The plot was obtained using the `plot_fitness` function. **C.** Theoretical and data-driven onset and duration of task events in the models. The R^2 shows the mean fitness of the models across the selected 80 ROIs. **D.** The evaluation of the models on six example ROIs (i.e., R_V1 – right primary visual cortex, R_V4 – right fourth visual area, R_FEF – right frontal eye fields, R_AIP – right anterior intraparietal area, R_POS1 – right parieto-occipital sulcus area 1, R_A1 – right primary auditory cortex; Glasser et al., 2016) with different types of BOLD response during a spatial working memory task. The plots were prepared using the `plot_model`, where the black line shows the average BOLD response, the pink line the modeled BOLD response and the thin colored lines depict individual responses to specific events.

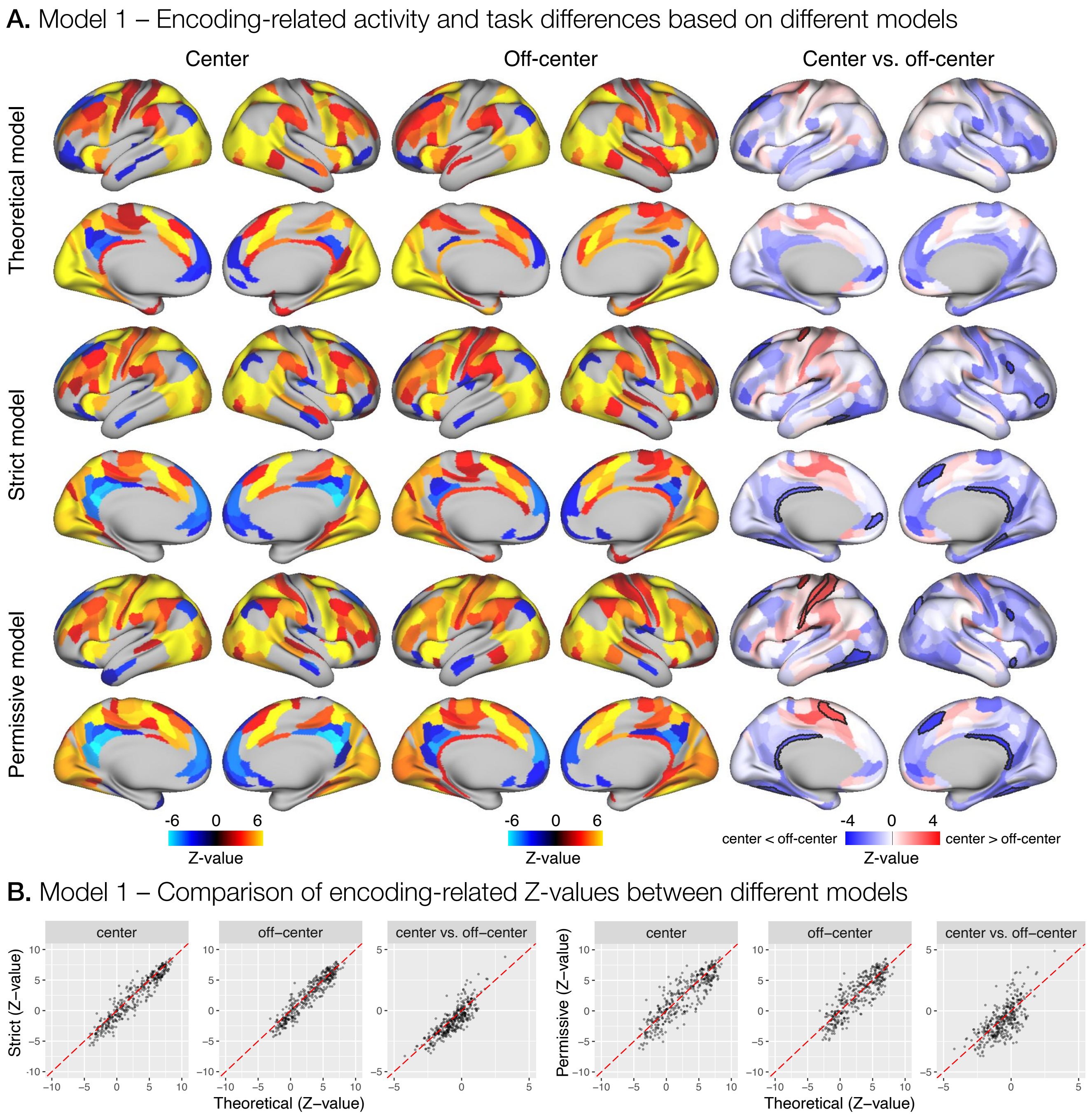

Figure S4. Encoding-related activity and differences between task conditions estimated based on different event models in the spatial working memory study. Results were obtained by modeling *encoding*, *delay*, and *response*, with the timing and duration of each event either theoretically determined or derived automatically based on the `autohrf` using less or more permissive constraints. **A.** Results of the statistical analysis show encoding-related activity separately for the *center* and *off-center* conditions and encoding-related activity differences between task conditions. The results of activation and deactivation during individual task conditions (the first two columns) show only statistically significant results at $q < 0.05$. On the other hand, the task differences (the third column) are presented across all ROIs, while the black outlines mark statistical significance at $q < 0.05$. **B.** The comparison of Z-values of individual ROIs between different models. Each dot represents the Z-value for a single parcel obtained with the contrasted models. The dashed red line represents the diagonal.

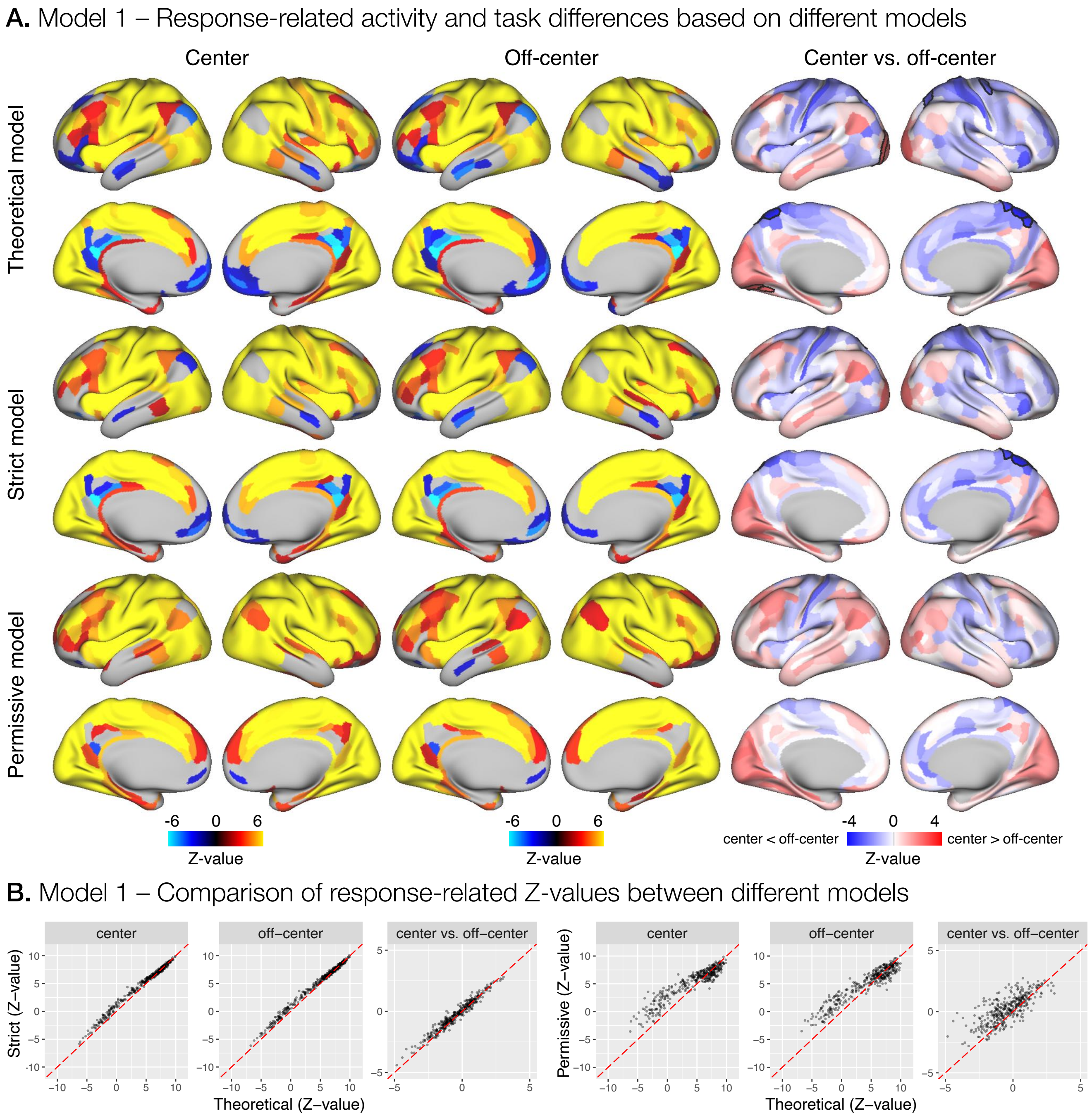

Figure S5. Response-related activity and differences between task conditions estimated based on different event models in the spatial working memory study. Results were obtained by modeling *encoding*, *delay*, and *response*, with the timing and duration of each event either theoretically determined or derived automatically based on the `autohrf` using less or more permissive constraints. **A.** Results of the statistical analysis show response-related activity separately for the *center* and *off-center* conditions and response-related activity differences between task conditions. The results of activation and deactivation during individual task conditions (the first two columns) show only statistically significant results at $q < 0.05$. On the other hand, the task differences (the third column) are presented across all ROIs, while the black outlines mark statistical significance at $q < 0.05$. **B.** The comparison of Z-values of individual ROIs between different models. Each dot represents the Z-value for a single parcel obtained with the contrasted models. The dashed red line represents the diagonal.

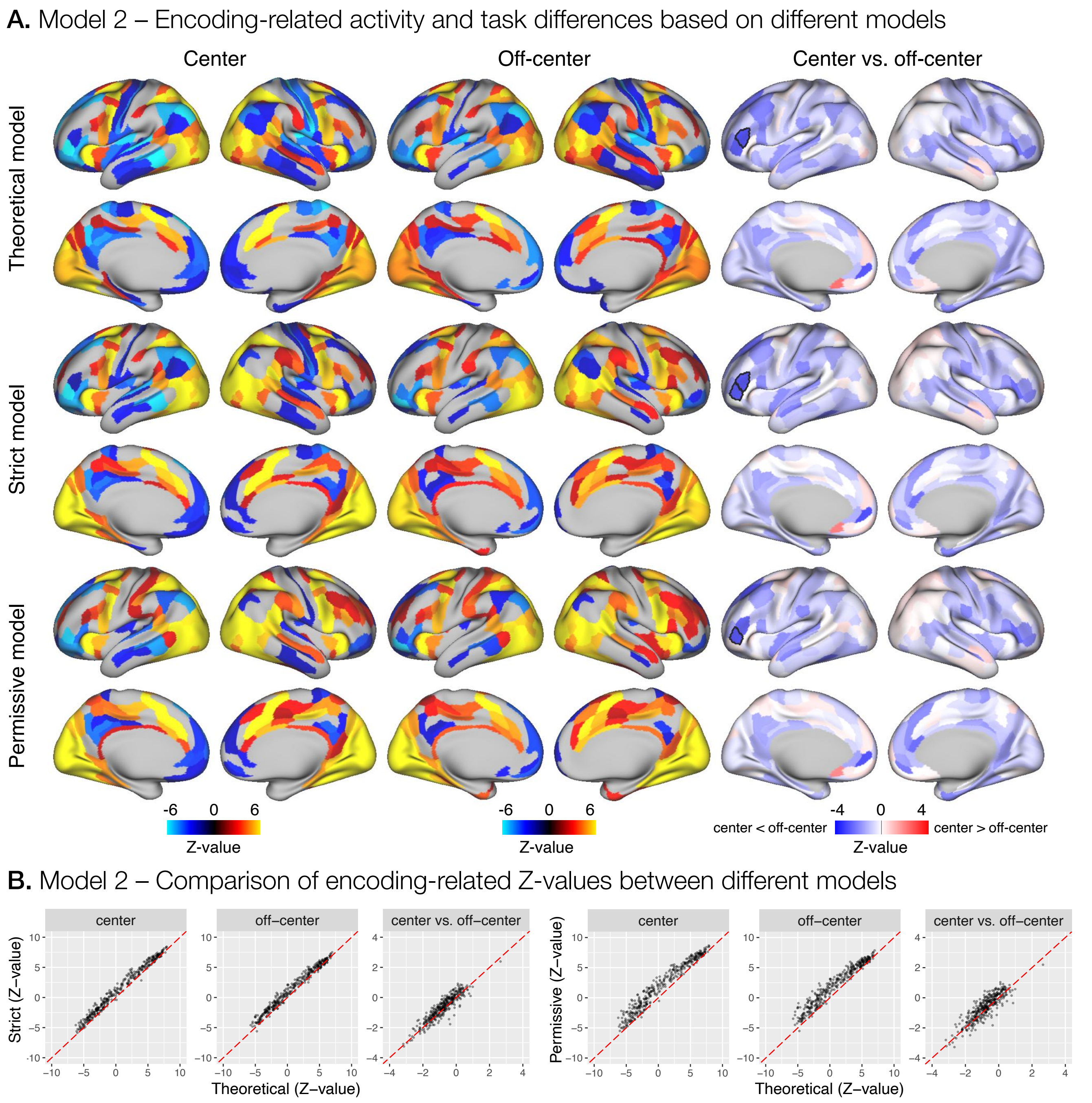

Figure S6. Encoding-related activity and differences between task conditions estimated based on different event models in the spatial working memory study. Results were obtained by modeling *encoding*, *early delay*, *late delay* and *response*, with the timing and duration of each event either theoretically determined or derived automatically based on the `autohrf` using less or more permissive constraints. **A.** Results of the statistical analysis show encoding-related activity separately for the *center* and *off-center* conditions and encoding-related activity differences between task conditions. The results of activation and deactivation during individual task conditions (the first two columns) show only statistically significant results at $q < 0.05$. On the other hand, the task differences (the third column) are presented across all ROIs, while the black outlines mark statistical significance at $q < 0.05$. **B.** The comparison of Z-values of individual ROIs between different models. Each dot represents the Z-value for a single parcel obtained with the contrasted models. The dashed red line represents the diagonal.

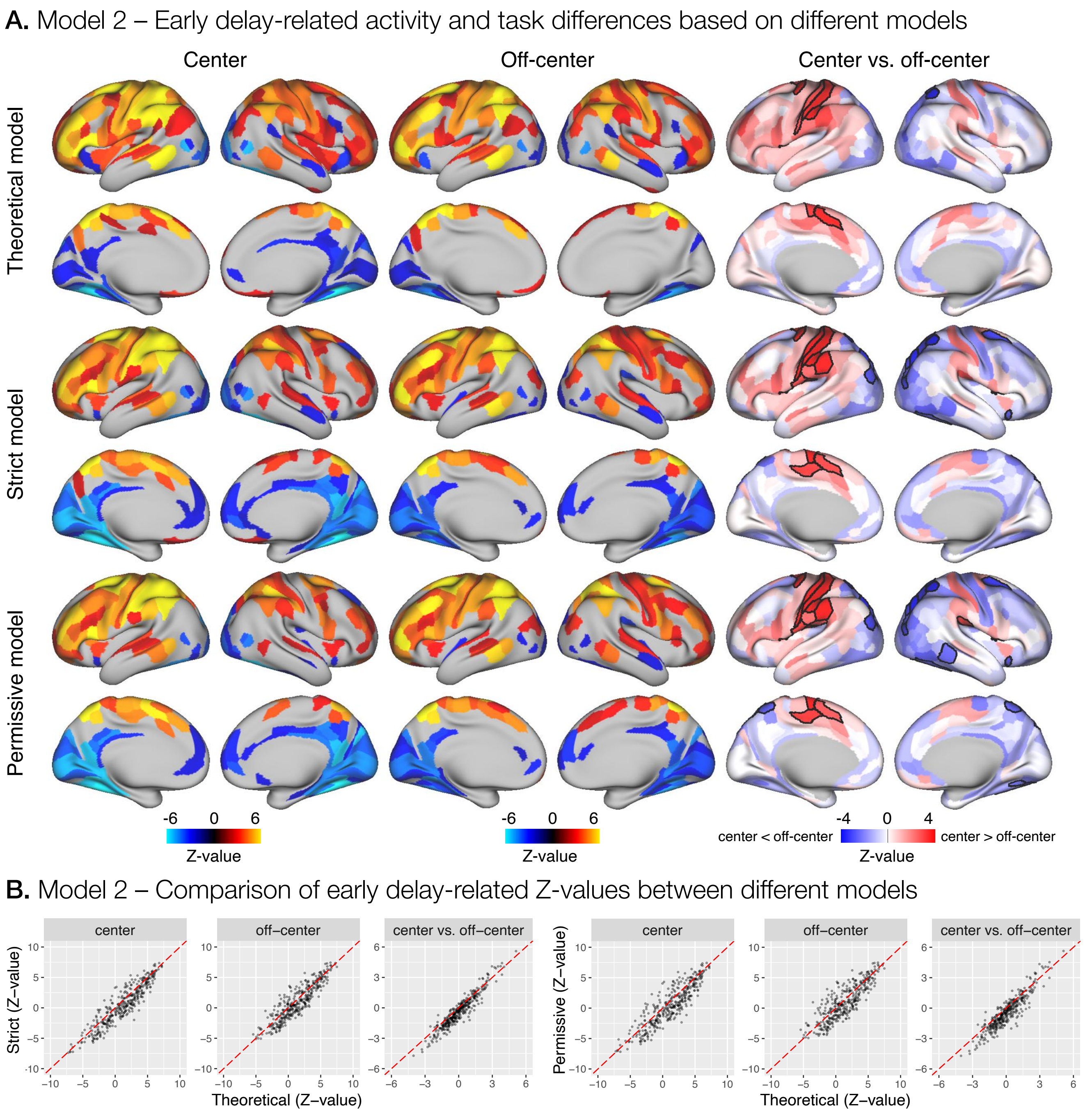

Figure S7. Early delay-related activity and differences between task conditions estimated based on different event models in the spatial working memory study. Results were obtained by modeling *encoding*, *early delay*, *late delay* and *response*, with the timing and duration of each event either theoretically determined or derived automatically based on the `autohrf` using less or more permissive constraints. **A.** Results of the statistical analysis show early delay-related activity separately for the *center* and *off-center* conditions and early delay-related activity differences between task conditions. The results of activation and deactivation during individual task conditions (the first two columns) show only statistically significant results at $q < 0.05$. On the other hand, the task differences (the third column) are presented across all ROIs, while the black outlines mark statistical significance at $q < 0.05$. **B.** The comparison of Z-values of individual ROIs between different models. Each dot represents the Z-value for a single parcel obtained with the contrasted models. The dashed red line represents the diagonal.

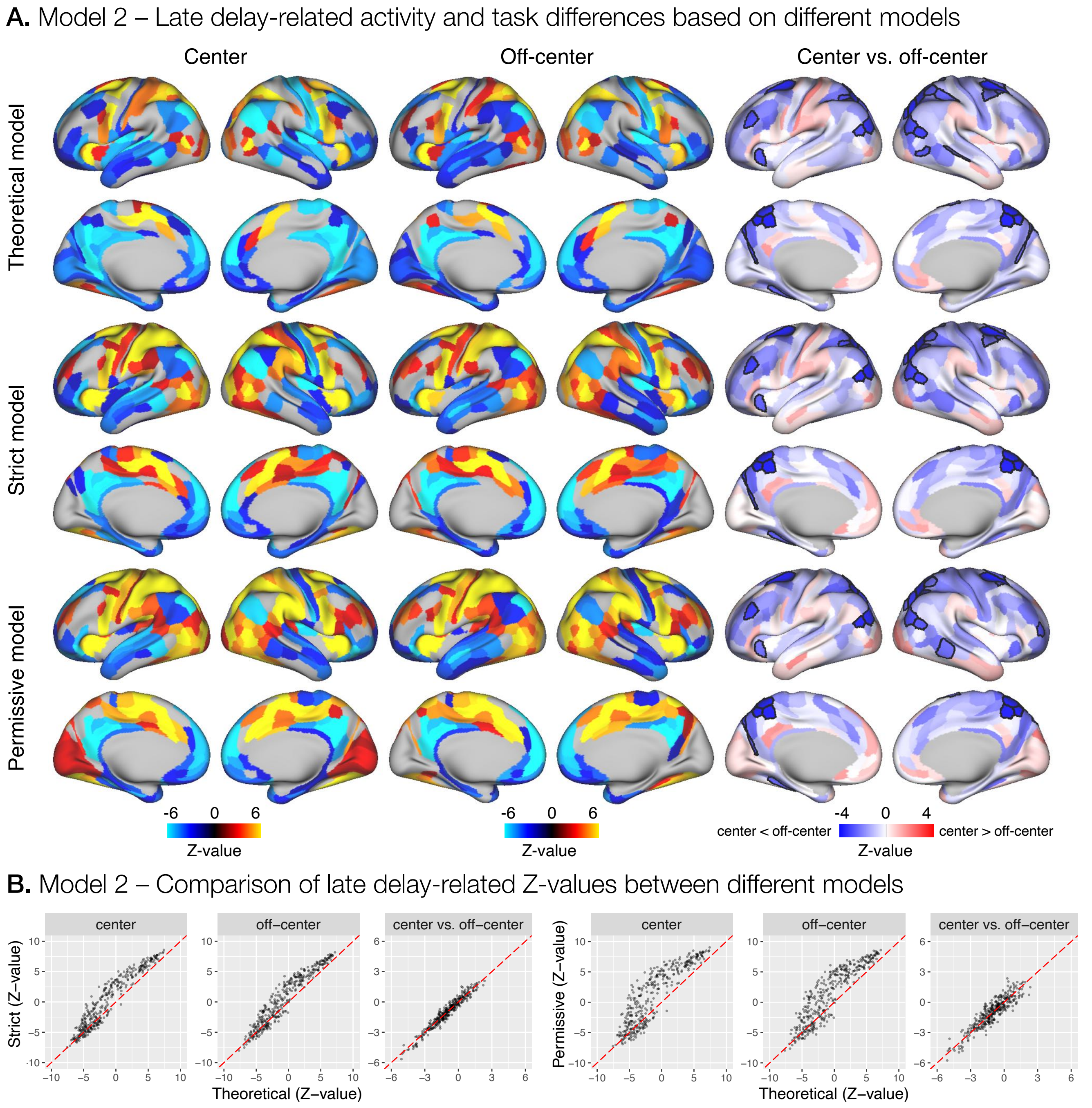

Figure S8. Late delay-related activity and differences between task conditions estimated based on different event models in the spatial working memory study. Results were obtained by modeling *encoding*, *early delay*, *late delay* and *response*, with the timing and duration of each event either theoretically determined or derived automatically based on the `autohrf` using less or more permissive constraints. **A.** Results of the statistical analysis show late delay-related activity separately for the *center* and *off-center* conditions and late delay-related activity differences between task conditions. The results of activation and deactivation during individual task conditions (the first two columns) show only statistically significant results at $q < 0.05$. On the other hand, the task differences (the third column) are presented across all ROIs, while the black outlines mark statistical significance at $q < 0.05$. **B.** The comparison of Z-values of individual ROIs between different models. Each dot represents the Z-value for a single parcel obtained with the contrasted models. The dashed red line represents the diagonal.

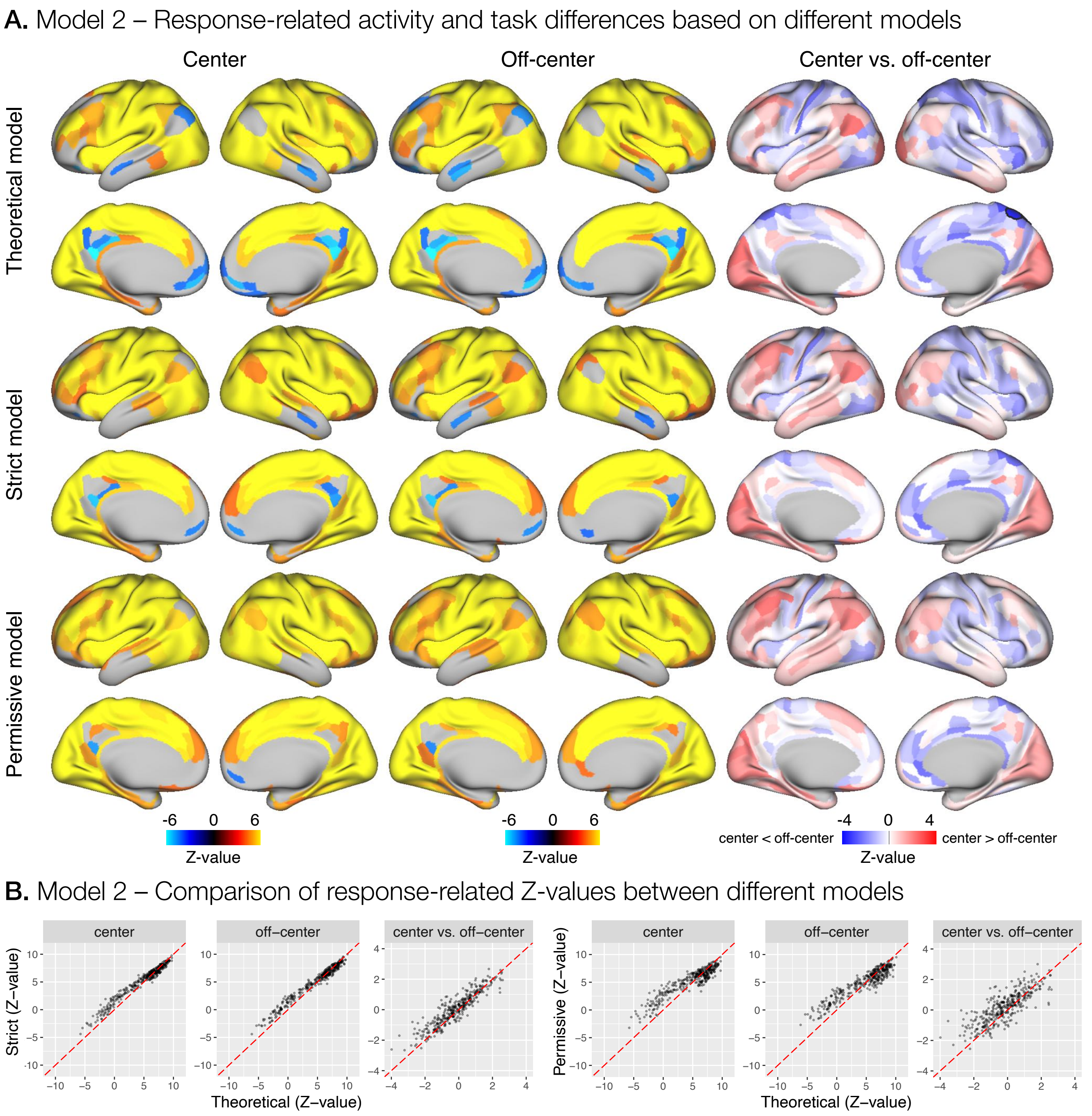

Figure S9. Response-related activity and differences between task conditions estimated based on different event models in the spatial working memory study. Results were obtained by modeling *encoding*, *early delay*, *late delay* and *response*, with the timing and duration of each event either theoretically determined or derived automatically based on the `autohrf` using less or more permissive constraints. **A.** Results of the statistical analysis show response-related activity separately for the *center* and *off-center* conditions and response-related activity differences between task conditions. The results of activation and deactivation during individual task conditions (the first two columns) show only statistically significant results at $q < 0.05$. On the other hand, the task differences (the third column) are presented across all ROIs, while the black outlines mark statistical significance at $q < 0.05$. **B.** The comparison of Z-values of individual ROIs between different models. Each dot represents the Z-value for a single parcel obtained with the contrasted models. The dashed red line represents the diagonal.

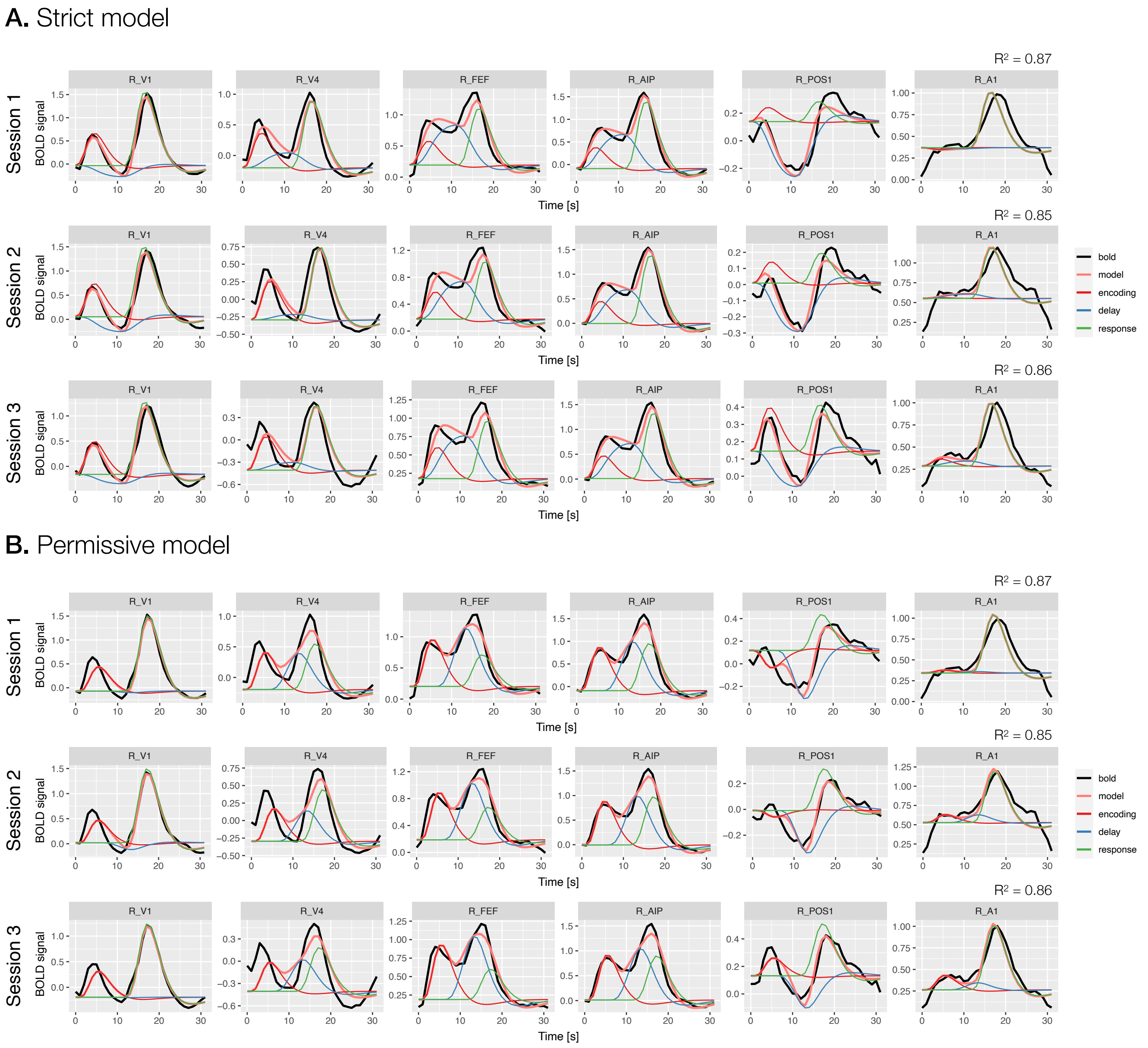

Figure S10. Cross-validation of automatically derived event models across different fMRI recording sessions in the spatial working memory study. Both models were obtained using `autohrf` based on the activity of 80 pre-selected ROIs in the frontoparietal, visual and motor-related brain areas during the first recording session, **A.** one with strict constraints and **B.** the other with more permissive constraints. Models were then evaluated using the `evaluate_model` based on the activity in the same selection of ROIs, but during second and third recording sessions. The plots were prepared using the `plot_model` for six example ROIs (i.e., R_V1 – right primary visual cortex, R_V4 – right fourth visual area, R_FEF – right frontal eye fields, R_AIP – right anterior intraparietal area, R_POS1 – right parieto-occipital sulcus area 1, R_A1 – right primary auditory cortex; Glasser et al., 2016) with different types of BOLD response during a spatial working memory task. The black line shows the average BOLD response, the pink line the modeled BOLD response and the thin colored lines depict individual responses to specific events. The R^2 shows the mean fitness of the models across the selected 80 ROIs.

A. Model parameters and fitness for different recording sessions

Session 1

model	event	start time	duration	fitness
strict	encoding	0.10	0.05	R^2 = 0.87
	delay	4.41	5.59	
	response	11.27	1.73	
permissive	encoding	0.64	0.25	R^2 = 0.89
	delay	6.00	5.00	
	response	11.14	2.86	

Session 2

model	event	start time	duration	fitness
strict	encoding	0.10	0.05	R^2 = 0.84
	delay	5.00	5.00	
	response	11.50	1.50	
permissive	encoding	0.46	0.51	R^2 = 0.87
	delay	6.00	5.00	
	response	11.73	2.27	

Session 3

model	event	start time	duration	fitness
strict	encoding	0.10	0.05	R^2 = 0.86
	delay	5.00	5.00	
	response	11.50	1.50	
permissive	encoding	0.50	0.47	R^2 = 0.89
	delay	6.00	5.00	
	response	11.39	2.61	

B. Overlap between model parameters across sessions

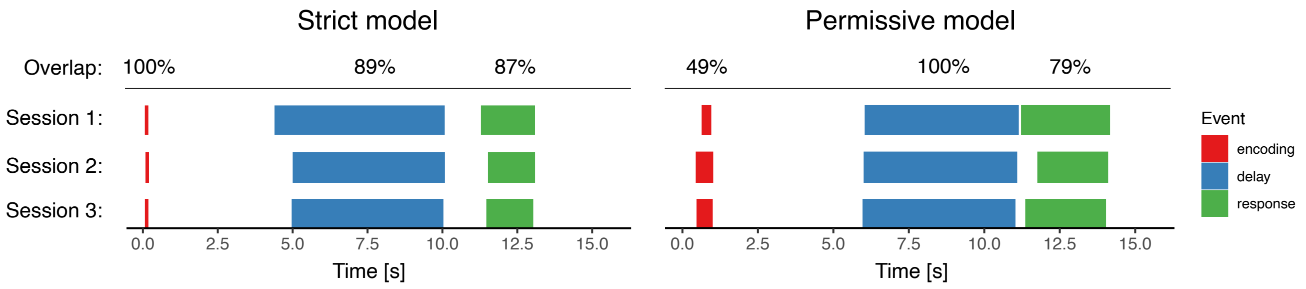

Figure S11. The comparison of automatically obtained event models based on different fMRI recording sessions in the spatial working memory study. Using `autohrf` function, we prepared two models based on the activity of 80 pre-selected ROIs in the frontoparietal, visual and motor-related brain areas, one with strict constraints and the other with more permissive constraints. Both models were run based on the fMRI signal obtained during separate recording sessions. **A.** Model parameters and fitness obtained for the strict and permissive model based on three fMRI recording sessions. The R^2 shows the mean fitness of the models across the selected 80 ROIs. **A.** The overlap in timing of specific events across different recording sessions. The overlap was computed as a percentage of time covered by a specific event in all recording sessions, in relation to the overall time interval that included the same event in at least one of the sessions.

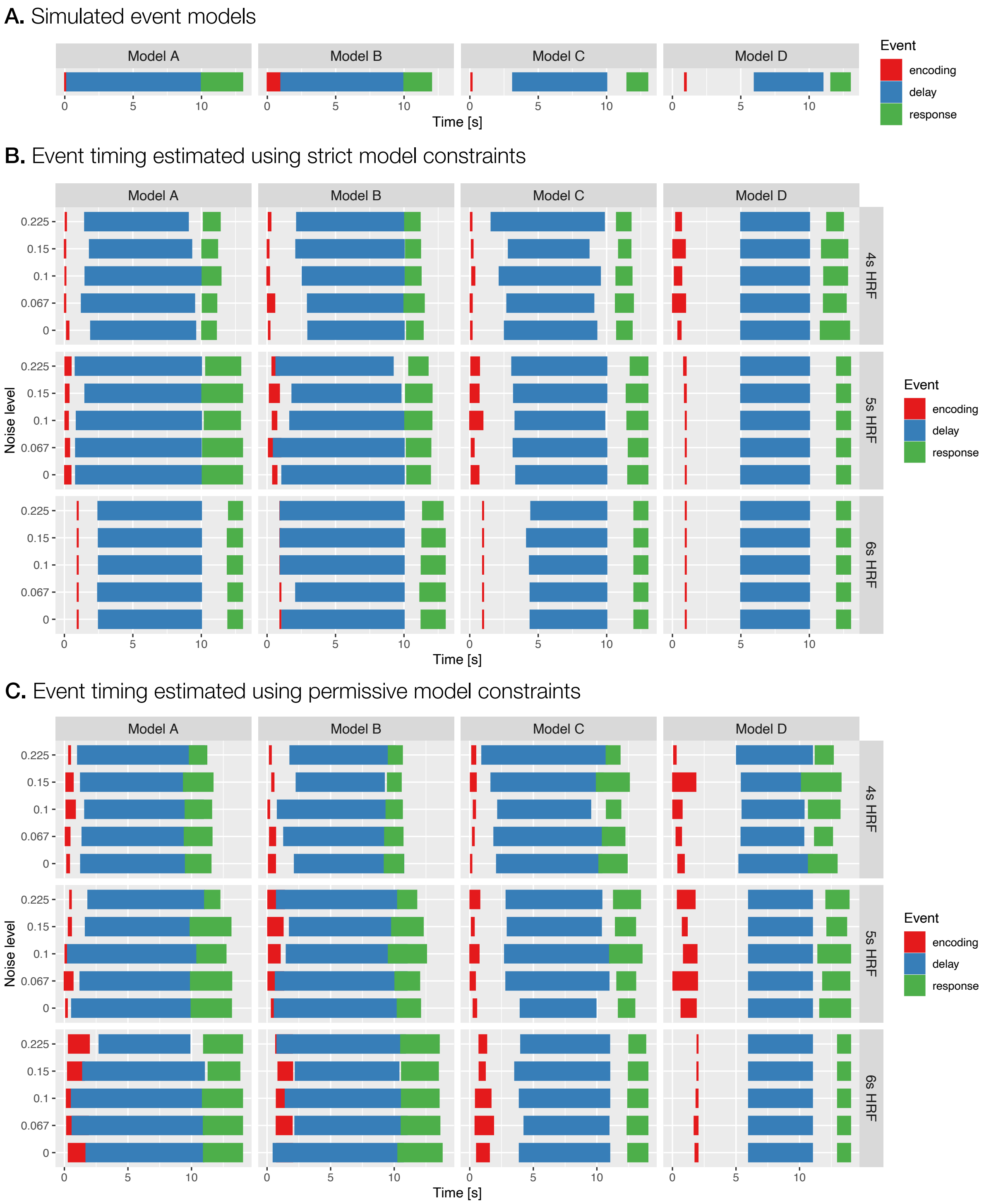

Figure S12. The comparison of simulated and automatically estimated event timing based on synthetic BOLD signal. A. The event onset and duration for four different models used in the simulation of fMRI data. All models included *encoding*, *delay*, and *response* events. **B.** The event onset and duration for *encoding*, *delay*, and *response* obtained using `autohrf` with strict constraints. The estimated event parameters are presented for four different simulated event models, three HRF times-to-peak, and five noise levels. **C.** The event onset and duration for *encoding*, *delay*, and *response* obtained using `autohrf` with permissive constraints. The estimated event parameters are presented for four different simulated event models, three HRF times-to-peak, and five noise levels.

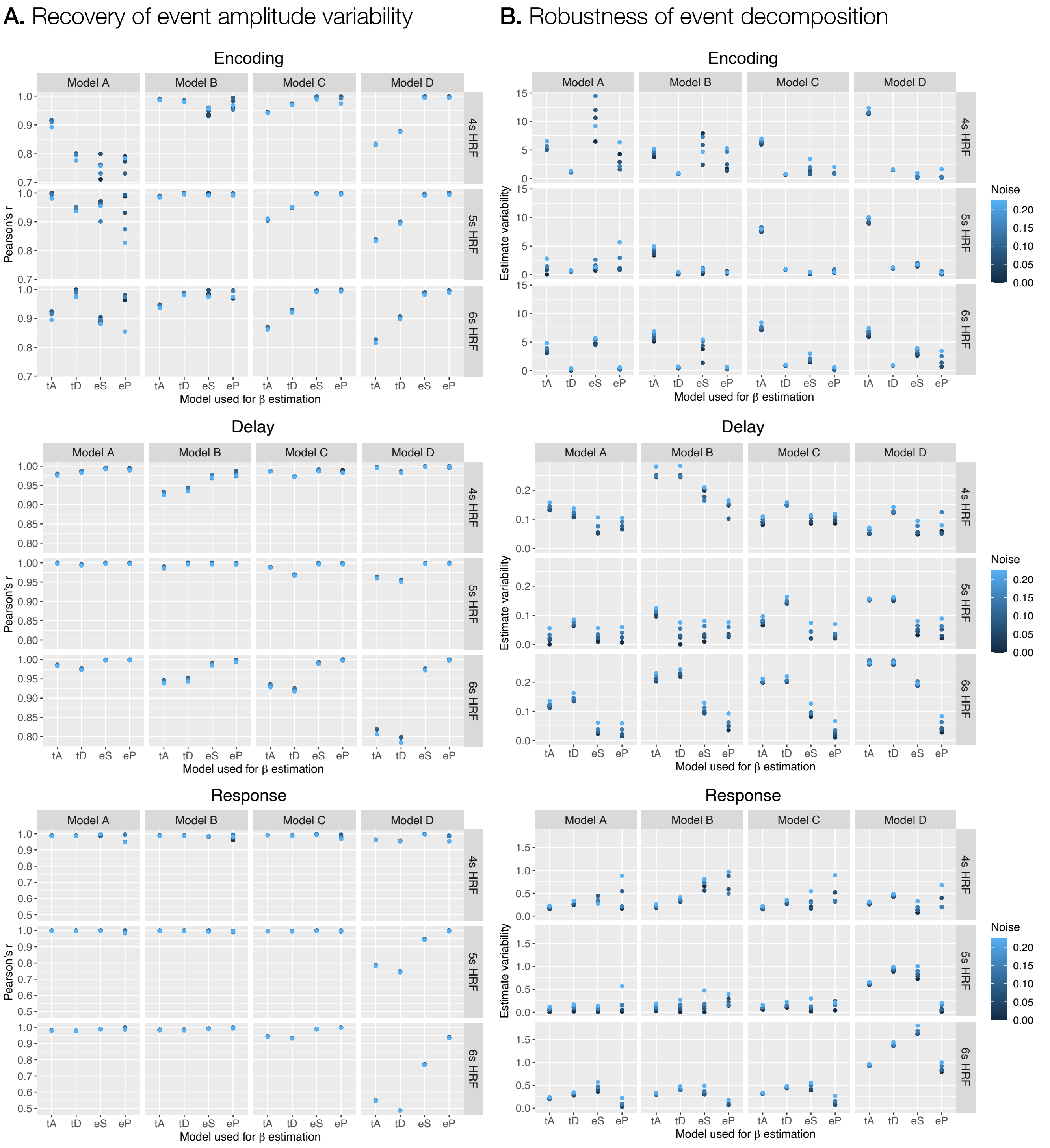

Figure S13. Evaluation of activation β estimates. A. Correlations between the simulated neural activity and estimated β values computed for each combination of event models used to generate the data, HRFs, noise levels, and event models used to estimate activity. **B.** Mean spans of β estimates across simulated ROI with the same activity value of the target event computed for each combination of models used to generate the data, HRFs, noise levels, and event models used to estimate activity. tA–theoretical model A, tB–theoretical model B, eS–empirically optimized models using strict constraints, eP–empirically optimized models using permissive constraints.

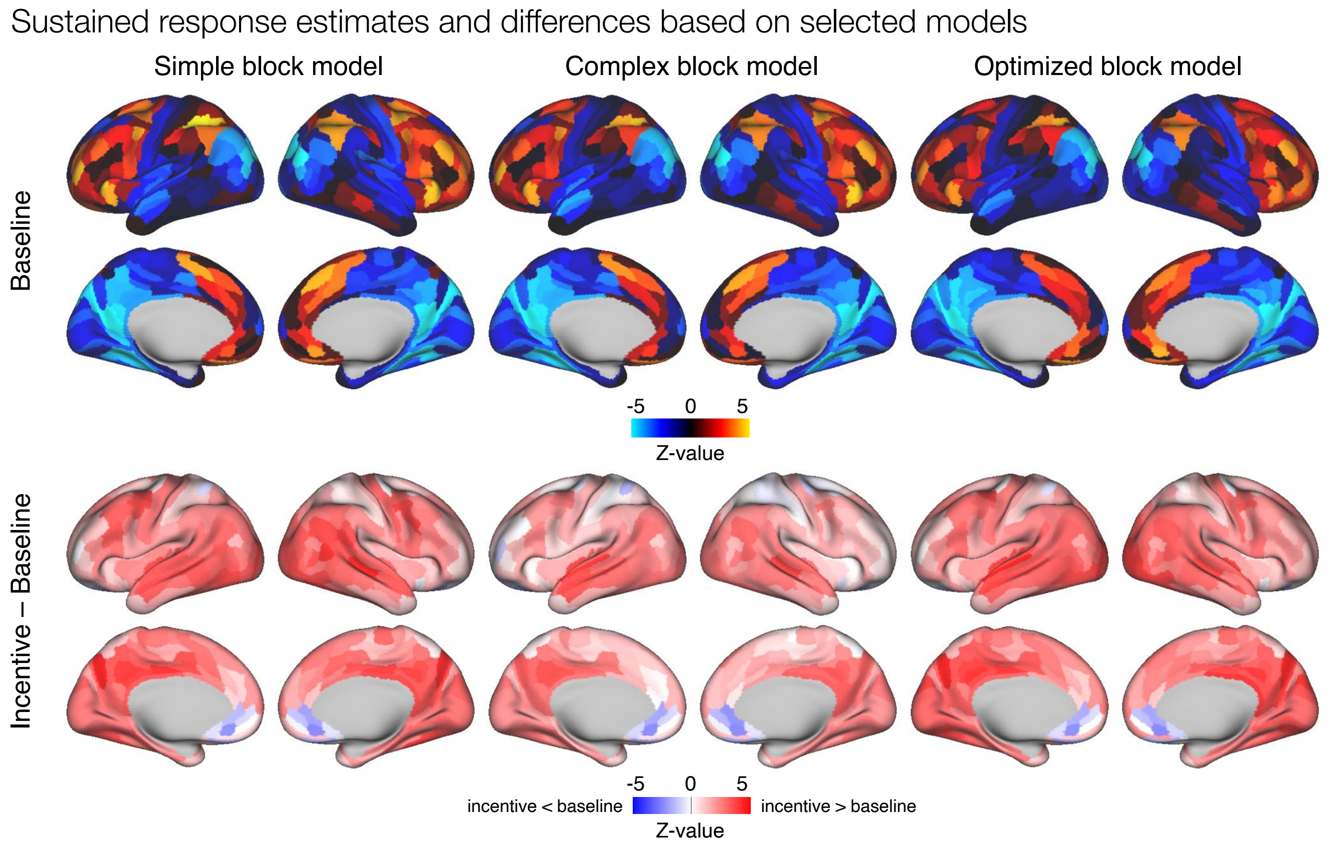

Figure S14. Estimation of task sustained response and incentive effects using different event models in the flanker study. Un-thresholded Z-values of whole-brain activations and deactivations in baseline condition (first row) and un-thresholded Z-values of differences between sustained task response in baseline and incentive conditions (second row) are shown.

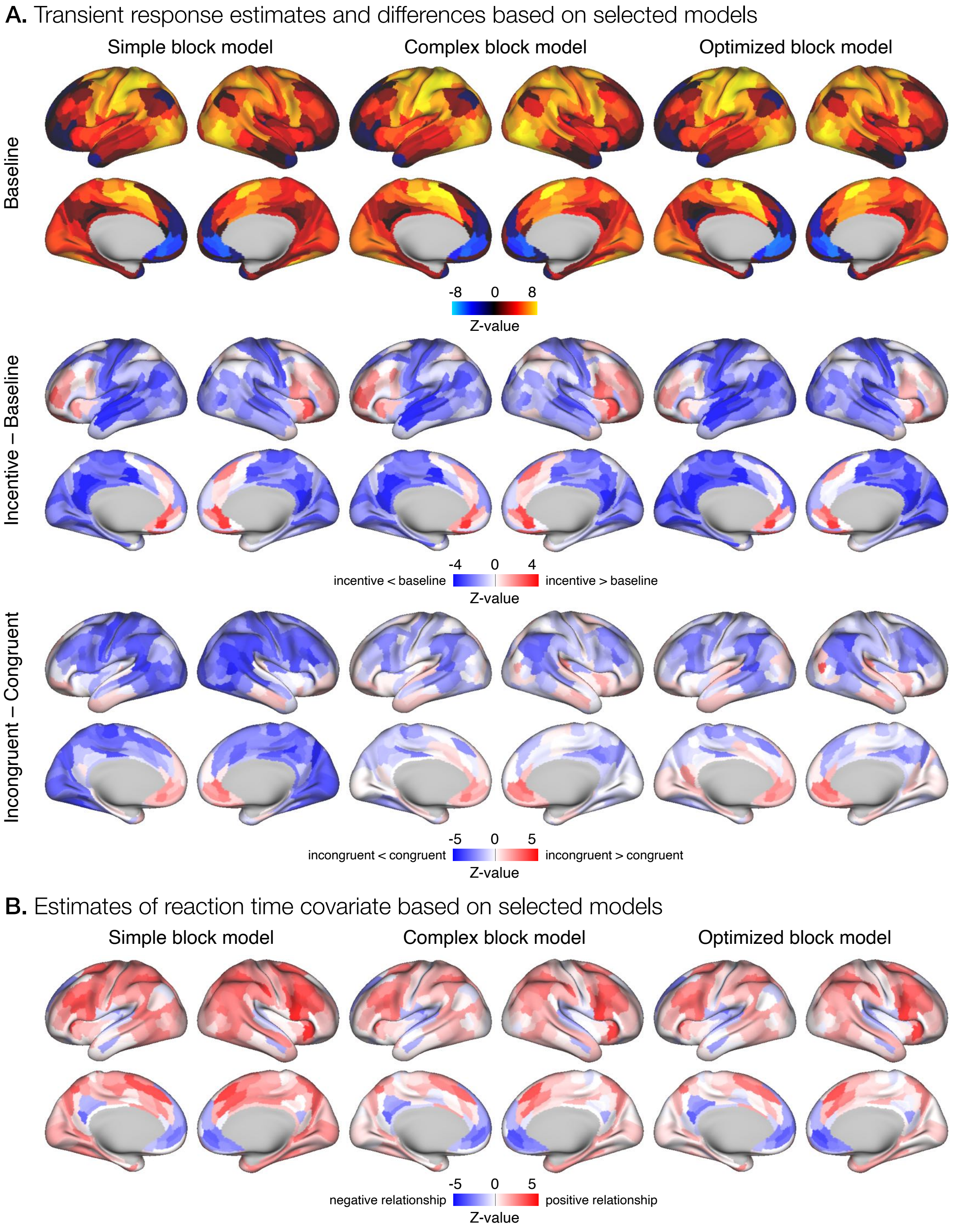

Figure S15. Estimation of transient task response, incentive and stimulus congruency effects, and reaction time covariation using different event models in the flanker study. A. Un-thresholded Z-values of estimates of transient whole-brain activations and deactivations in the baseline condition (top row), differences in transient response between incentive and baseline conditions (middle row), and differences between responses to incongruent and congruent stimuli in baseline condition (bottom row) are shown. **B.** Un-thresholded whole-brain maps of Z-values of β estimates reflecting covariability between trial-to-trial transient responses and reaction times are shown.

REFERENCES

Aguirre, G., Zarahn, E., and D'Esposito, M. (1998). The Variability of Human, BOLD Hemodynamic Responses. *NeuroImage* 8, 360–369. doi:10.1006/nimg.1998.0369

Boynton, G. M., Engel, S. A., Glover, G. H., and Heeger, D. J. (1996). Linear Systems Analysis of Functional Magnetic Resonance Imaging in Human V1. *The Journal of Neuroscience* 16, 4207–4221. doi:10.1523/JNEUROSCI.16-13-04207.1996

Friston, K., Fletcher, P., Josephs, O., Holmes, A., Rugg, M., and Turner, R. (1998). Event-Related fMRI: Characterizing Differential Responses. *NeuroImage* 7, 30–40. doi:10.1006/nimg.1997.0306

Friston, K. J., Holmes, A. P., Worsley, K. J., Poline, J.-P., Frith, C. D., and Frackowiak, R. S. J. (1994). Statistical parametric maps in functional imaging: A general linear approach. *Human Brain Mapping* 2, 189–210. doi:10.1002/hbm.460020402

Glasser, M. F., Coalson, T. S., Robinson, E. C., Hacker, C. D., Harwell, J., Yacoub, E., et al. (2016). A multi-modal parcellation of human cerebral cortex. *Nature* 536, 171–178. doi:10.1038/nature18933

Glasser, M. F., Sotiropoulos, S. N., Wilson, J. A., Coalson, T. S., Fischl, B., Andersson, J. L., et al. (2013). The minimal preprocessing pipelines for the Human Connectome Project. *NeuroImage* 80, 105–124. doi:10.1016/j.neuroimage.2013.04.127

Handwerker, D. A., Ollinger, J. M., and D'Esposito, M. (2004). Variation of BOLD hemodynamic responses across subjects and brain regions and their effects on statistical analyses. *NeuroImage* 21, 1639–1651. doi:10.1016/j.neuroimage.2003.11.029

Ji, J. L., Demšar, J., Fonteneau, C., Tamayo, Z., Pan, L., Kraljič, A., et al. (2022). QuNex – An Integrative Platform for Reproducible Neuroimaging Analytics. *bioRxiv* doi:10.1101/2022.06.03.494750

Miezin, F., Maccotta, L., Ollinger, J., Petersen, S., and Buckner, R. (2000). Characterizing the Hemodynamic Response: Effects of Presentation Rate, Sampling Procedure, and the Possibility of Ordering Brain Activity Based on Relative Timing. *NeuroImage* 11, 735–759. doi:10.1006/nimg.2000.0568
